# Diagnostic Accuracy of VE1 Immunohistochemistry for BRAF p.V600E in Colorectal Carcinoma: A Systematic Review

**DOI:** 10.3390/medicina62101974

**Published:** 2026-10-12

**Authors:** Ovidiu-Ioan Șușu, Marian Botoncea, Gabriel Serac, Marius-Alexandru Beleaua, Tamás-Csaba Sipos, Oana Alexandra Tăurean, Dragoș Călin Molnar, Remus-Sebastian Șipoș, Zsuzsánna Pap

**Affiliations:** 1 Faculty of Medicine, George Emil Palade University of Medicine, Pharmacy, Sciences and Technology of Târgu Mureș, 38 Gheorghe Marinescu Street, 540139 Târgu Mureș, Romania; ovidiu.susu@umfst.ro (O.-I.Ș.); marius.beleaua@umfst.ro (M.-A.B.); tamas.sipos@umfst.ro (T.-C.S.); oanataurean@yahoo.com (O.A.T.); remus.sipos@umfst.ro (R.-S.Ș.); zsuzsanna.pap@umfst.ro (Z.P.); 2 General Surgery Clinic No. 1, County Emergency Clinical Hospital of Târgu Mureș, 50 Gheorghe Marinescu Street, 540136 Târgu Mureș, Romania; gabiserac@gmail.com (G.S.); dragosmolnar2000@yahoo.com (D.C.M.); 3 Doctoral School of Medicine and Pharmacy, George Emil Palade University of Medicine, Pharmacy, Sciences and Technology of Târgu Mureș, 540142 Târgu Mures, Romania; 4 Pathology Department, County Emergency Clinical Hospital of Bistrița, 43 General Grigore Bălan Boulevard, 420094 Bistrița-Năsăud, Romania; 5 Pathology Department, County Emergency Clinical Hospital of Târgu Mureș, 50 Gheorghe Marinescu Street, 540136 Târgu Mureș, Romania

**Keywords:** BRAF V600E, colorectal cancer, immunohistochemistry, VE1 antibody, molecular testing, diagnostic concordance, mismatch repair deficiency, Lynch syndrome

## Abstract

*Background and Objectives*: BRAF p.V600E status has diagnostic, prognostic, and therapeutic relevance in colorectal carcinoma. Molecular assays remain the reference methods, whereas mutation-specific BRAF (VE1 clone) immunohistochemistry offers a rapid tissue-based alternative. We evaluated VE1–molecular concordance and factors affecting diagnostic performance. *Materials and Methods*: PubMed/MEDLINE, Scopus, and Web of Science were searched in August 2025 and updated on 27 July 2026 for English-language original studies published from 2014 through the update date. Eligible reports applied VE1 and a recognized molecular reference method to the same colorectal carcinoma specimens or a defined overlapping cohort. Study selection and data extraction were performed by one reviewer and independently verified by a second reviewer. Risk of bias and applicability were assessed at the estimate level with QUADAS-3. Because of substantial clinical and methodological heterogeneity, no meta-analysis was performed, and the findings were synthesized descriptively. *Results*: Of 704 database records, 358 unique records were screened; 38 full-text reports were assessed and 28 were included. Twenty-three reports or defined subcohorts provided complete or clearly reconstructible 2 × 2 comparisons. Sensitivity ranged from 74.6% to 100% and specificity from 66.7% to 100%. Most optimized protocols exceeded 90% for both measures, whereas selected sampling, weak or focal staining, post hoc thresholds, protocol effects, and selective molecular verification reduced reliability. *Conclusions*: VE1 immunohistochemistry may provide a rapid, locally validated triage method for detecting BRAF p.V600E in colorectal carcinoma. However, substantial heterogeneity and recurrent risk-of-bias concerns limit the generalizability of the available estimates and do not support universal substitution for molecular testing. Equivocal, technically inadequate, or clinically discordant results require molecular clarification.

## 1. Introduction

Colorectal cancer (CRC) is one of the most common malignancies of the gastrointestinal tract and remains a major cause of cancer-related morbidity and mortality worldwide [1,2,3,4]. It is among the most frequently diagnosed cancers in both men and women and continues to represent a substantial global health burden despite advances in screening, diagnosis, and treatment [5,6]. Recent epidemiological data have also demonstrated an increasing incidence of colorectal cancer among younger individuals, particularly those diagnosed before the age of 50 years, emphasizing the need for improved diagnostic and prognostic biomarkers [7,8].

Among the molecular alterations involved in colorectal carcinogenesis, mutations of the BRAF gene have emerged as clinically relevant biomarkers. BRAF encodes a serine-threonine kinase that functions within the RAS–RAF–MEK–ERK/MAPK signaling pathway, a key regulator of cellular proliferation, differentiation, and apoptosis [9,10,11]. The most common BRAF alteration in colorectal carcinoma is the V600E mutation, resulting from the substitution of valine by glutamic acid at codon 600. This mutation leads to constitutive activation of the MAPK pathway and promotes uncontrolled cellular growth independently of physiological regulatory mechanisms [10,12,13,14]. BRAF mutations occur in approximately 8–15% of colorectal carcinomas, with the V600E variant accounting for most cases [10,12,15,16].

Numerous studies have demonstrated that BRAF V600E is associated with adverse clinicopathological characteristics and poorer clinical outcomes in colorectal cancer [4,11,14]. In addition to its prognostic significance, BRAF mutation status has important predictive implications, particularly in metastatic colorectal cancer, where it may influence therapeutic decision-making and response to anti-EGFR-based treatment strategies [3,7,8,14,17].

The identification of BRAF V600E also plays an important role in the diagnostic evaluation of mismatch repair-deficient (dMMR) and microsatellite instability-high (MSI-H) colorectal carcinomas. In tumors demonstrating loss of MLH1/PMS2 expression, the presence of a BRAF V600E mutation strongly supports a sporadic pathway of carcinogenesis associated with MLH1 promoter hypermethylation and reduces the likelihood of Lynch syndrome [9,18,19,20,21,22]. However, the absence of a BRAF mutation does not exclude Lynch syndrome and should always be interpreted in conjunction with mismatch repair status, MLH1 promoter methylation analysis, and the clinical context.

Molecular techniques such as Sanger sequencing, PCR-based assays, pyrosequencing, and next-generation sequencing are considered reference methods for the detection of BRAF mutations. Although these methods provide excellent analytical accuracy, they require specialized laboratory infrastructure, increase diagnostic costs, and may prolong turnaround times [11,19]. Consequently, immunohistochemistry (IHC) has attracted considerable interest as a potential screening alternative in routine pathology practice.

BRAF V600E immunohistochemical detection is most commonly performed using the mutation-specific monoclonal antibody VE1, which recognizes the mutant BRAF V600E protein and produces a characteristic cytoplasmic staining pattern in tumor cells [15,18,21]. Because VE1 is specific for the V600E mutant protein, it does not provide comprehensive BRAF genotyping and cannot exclude non-V600E BRAF variants. Several studies have reported high sensitivity and specificity for VE1 immunohistochemistry, suggesting that it may represent a rapid, cost-effective, and widely accessible approach for identifying BRAF-mutated colorectal carcinomas [15,19,21]. Nevertheless, published results remain heterogeneous, with reported differences in diagnostic accuracy, interpretation criteria, staining protocols, and concordance with molecular testing [10,20,23,24].

Given the increasing clinical relevance of BRAF V600E status in colorectal carcinoma and the expanding use of immunohistochemistry in routine pathology practice, a comprehensive evaluation of its diagnostic performance is warranted. Therefore, the aim of the present review was to analyze the concordance between BRAF V600E immunohistochemistry and molecular testing in colorectal carcinoma, to evaluate the diagnostic accuracy of IHC across different study populations and methodologies, and to identify technical and interpretative factors that may influence agreement between immunohistochemical and molecular results.

A previous diagnostic meta-analysis by Pyo et al. included eight studies and reported high pooled diagnostic performance for VE1 immunohistochemistry [16]. However, that analysis preceded several larger routine-practice cohorts and did not explore in detail differences in staining protocols, interpretation thresholds, equivocal staining, or selective molecular verification. The present review therefore updates the evidence and focuses on the methodological and technical factors affecting the reproducibility of VE1 performance in routine clinical practice, while incorporating estimate-level QUADAS-3 [25] assessment.

## 2. Materials and Methods

### 2.1. Study Design, Protocol Amendment, and Reporting Standard

This systematic review evaluated the diagnostic concordance and accuracy of BRAF V600E immunohistochemistry compared with molecular testing in colorectal carcinoma. The review was initiated in August 2025 with an intended decade-long scope. Before the review was finalized, the search was updated, and the publication window was expanded to 1 January 2014 through 27 July 2026 to retain early VE1 validation studies and incorporate newly available evidence. This change is reported as a methodological amendment because the protocol was not prospectively registered. The absence of prospective registration limits the ability to distinguish fully prespecified decisions from modifications introduced during review conduct and therefore represents a potential source of methodological bias. The review was conducted and reported in accordance with the Preferred Reporting Items for Systematic Reviews and Meta-Analyses 2020 statement (PRISMA 2020) [26], and the diagnostic-test approach was informed by the PRISMA-DTA statement [27]. The completed PRISMA 2020 checklist is available in the Supplementary Materials (PRISMA 2020 Checklist).

### 2.2. Information Sources and Search Strategy

Systematic searches were performed in PubMed/MEDLINE, Scopus, and Web of Science Core Collection. The initial searches were conducted in August 2025. An updated search using the same conceptual framework was completed on 27 July 2026 to identify records added after the initial search and to finalize the evidence base.

The final eligibility window covered articles published from 1 January 2014 through 27 July 2026. The search strategy combined controlled vocabulary terms, where available, with free-text terms related to colorectal carcinoma, BRAF V600E, VE1 or mutation-specific immunohistochemistry, diagnostic concordance, and molecular testing. The database-specific strategies reported below correspond to the final search framework.

The main PubMed/MEDLINE search strategy was:

((“Colorectal Neoplasms”[Mesh] OR “colorectal cancer”[Title/Abstract] OR “colorectal cancers”[Title/Abstract] OR “colorectal carcinoma”[Title/Abstract] OR “colorectal carcinomas”[Title/Abstract] OR “colon cancer”[Title/Abstract] OR “colon carcinoma”[Title/Abstract] OR “colonic carcinoma”[Title/Abstract] OR “rectal cancer”[Title/Abstract] OR “rectal carcinoma”[Title/Abstract]) AND (“Proto-Oncogene Proteins B-raf”[Mesh] OR BRAF[Title/Abstract] OR “BRAF V600E”[Title/Abstract] OR “BRAF-V600E”[Title/Abstract] OR BRAFV600E[Title/Abstract] OR “V600E mutation”[Title/Abstract]) AND (“Immunohistochemistry”[Mesh] OR immunohistochem*[Title/Abstract] OR IHC[Title/Abstract] OR VE1[Title/Abstract] OR “VE1 antibody”[Title/Abstract] OR “mutation-specific antibody”[Title/Abstract])).

For the Scopus database, the following query was used:

“TITLE-ABS-KEY ((“colorectal cancer” OR “colorectal carcinoma” OR “colon cancer” OR “colon carcinoma” OR “rectal cancer” OR “rectal carcinoma”) AND (“BRAF V600E” OR “BRAF-V600E” OR BRAFV600E OR (BRAF AND V600E)) AND (immunohistochem* OR IHC OR VE1 OR “VE1 antibody”) AND (concordan* OR sensitiv* OR specificit* OR accurac* OR molecular OR sequencing OR PCR OR pyrosequencing OR NGS)) AND (LIMIT-TO (LANGUAGE, “English”)) AND PUBYEAR > 2013 AND PUBYEAR < 2027”.

For Web of Science the following search strategy was used:

“ TS = ((“colorectal cancer” OR “colorectal carcinoma” OR “colon cancer” OR “colon carcinoma” OR “rectal cancer” OR “rectal carcinoma”) AND (“BRAF V600E” OR “BRAF-V600E” OR BRAFV600E OR (BRAF AND V600E)) AND (immunohistochem* OR IHC OR VE1) AND (concordan* OR accurac* OR sensitiv* OR specificit* OR molecular OR sequencing OR PCR OR pyrosequencing OR NGS))”.

Only full-text original articles published in English were considered eligible. Reference lists of included studies and relevant reviews were screened manually. Results from the August 2025 searches and the July 2026 update were combined before final deduplication and screening.

### 2.3. Eligibility Criteria

Eligible studies were English-language full-text original research articles published within the specified window and involving at least four colorectal carcinoma cases. They had to evaluate BRAF V600E protein expression using the mutation-specific VE1 monoclonal antibody and assess BRAF mutation status using a recognized molecular reference method. Both tests had to be applied to the same study population or a clearly defined overlapping cohort, with numerical data permitting comparison of immunohistochemical and molecular results.

Reviews, systematic reviews, meta-analyses, editorials, letters, conference abstracts, case reports, and methodological articles without clinical validation were excluded, as were studies conducted exclusively in cell lines, animal models, or non-human tissue. Studies were also excluded if they did not specifically evaluate colorectal carcinoma, used an antibody clone other than VE1, primarily investigated other biomarkers without separately extractable BRAF V600E data, did not specify the molecular reference method, applied the two tests to non-comparable cohorts, or provided insufficient numerical data to assess diagnostic concordance.

The review focused on primary colorectal carcinoma specimens. Studies predominantly assessing metastatic lesions were excluded unless primary colorectal carcinomas were adequately represented or their results were separately extractable. For duplicate publications or overlapping patient cohorts, the most complete or most recent dataset was retained.

The restriction regarding predominantly metastatic cohorts reflected the predefined focus of this review on diagnostic performance in primary colorectal carcinoma specimens; studies including metastatic tissue remained eligible when results for primary colorectal carcinoma were adequately represented or separately extractable.

### 2.4. Study Selection

All retrieved records were imported into Zotero version 9.0.6 reference-management software, and duplicate records were removed before screening. Detailed search, screening, retrieval, and inclusion counts are presented in Supplementary Table S1.

Title/abstract screening began after the August 2025 searches. Records identified by the July 2026 update were screened using the same final eligibility criteria. O.I.Ș. performed the title/abstract and full-text screening, and G.S. independently verified all decisions against the source records. Screening decisions were recorded in a structured audit log.

For every report assessed in full text, O.I.Ș. recorded the eligibility decision and one primary exclusion reason. G.S. independently verified the report-level decisions and supporting evidence. Any differences were resolved through discussion and consensus. When consensus could not be reached, a third reviewer (M.B.) was consulted.

The study-selection process was documented using a PRISMA 2020 flow diagram. Reports that could not be obtained in full text were recorded separately as not retrieved and were not assessed for eligibility, extracted, or included in the risk-of-bias assessment. Full-text exclusions are reported using one primary eligibility reason; the report-level audit trail is provided in Supplementary Table S2. The reports sought but not retrieved are listed in Supplementary Table S3.

### 2.5. Data Extraction

Data were extracted by O.I.Ș. using a predefined standardized form and independently verified against the source reports by M.B. No diagnostic data were extracted from reports that were not retrieved. Discrepancies, including equivocal classifications and selectively verified cohorts, were resolved by re-examination of the source report and consensus.

For each eligible study, the extracted information included first author, publication year, country, design, setting, patient-selection method, total cohort size, and the number assessed by both tests. Population characteristics included whether the cohort was unselected, mismatch repair-deficient, microsatellite instability-high, or MLH1-deficient. Tissue type and format were recorded, including whole-tissue sections, tissue microarrays, biopsies, and metastatic specimens.

Technical data included the antibody clone, source and dilution, staining platform, antigen retrieval, detection procedures, staining pattern and intensity, positivity threshold, definition and management of equivocal staining, and the number, experience, and blinding of readers. Pre-analytical and processing details included cold ischemia interval, fixative type or buffering, and fixation duration when reported. The molecular reference method, mutation spectrum, molecular-verification strategy, and procedures for resolving discordance were also extracted. Characteristics not explicitly reported in the original article were recorded as not reported and were not inferred.

Diagnostic data comprised the numbers of BRAF V600E-positive and wild-type cases; true-positive, false-positive, false-negative, and true-negative results when derivable; and reported sensitivity, specificity, positive and negative predictive values, concordance, and Cohen’s kappa. MLH1, mismatch repair, and microsatellite instability status and reported causes of immunohistochemical–molecular discordance were recorded.

Study-level technical characteristics were also extracted, including specimen type and format; pre-analytical and fixation/processing details, including cold ischemia interval, fixative type or buffering, and fixation duration when reported; VE1 antibody source and dilution; staining platform; antigen-retrieval and detection procedures; interpretation threshold; definition and management of equivocal staining; number and blinding of readers; molecular reference method; mutation spectrum evaluated; molecular-verification strategy; and procedures used to resolve discordant results. Characteristics not explicitly reported in the original article were recorded as not reported and were not inferred.

### 2.6. Data Synthesis

Because of substantial clinical and methodological heterogeneity in study populations, specimen types, staining platforms, positivity criteria, and molecular reference methods, findings were synthesized descriptively. Sensitivity was calculated as TP/(TP + FN) and specificity as TN/(TN + FP); two-sided 95% Clopper–Pearson exact confidence intervals were calculated for complete or clearly reconstructible matrices. Published handling of equivocal results was preserved whenever possible. For Boulagnon [9] and Vakiani [28], the available data permitted only a conservative sensitivity analysis in which molecular-positive equivocal results were treated as false negatives and molecular-negative equivocal results as false positives; these rows are explicitly flagged. Author-reported metrics were retained without invented cell counts when observer-specific values, equivocal categories, or published totals did not support a unique 2 × 2 table. No pooled diagnostic-accuracy estimate was calculated. The absence of complete or uniquely reconstructible 2 × 2 contingency tables in several reports represented an additional barrier to valid quantitative pooling. Rules for handling and reporting equivocal VE1 results are summarized in Supplementary Table S4.

Although 23 studies provided complete or clearly reconstructible 2 × 2 data, a meta-analysis was not performed because the studies did not estimate a sufficiently comparable diagnostic target. Substantial clinical and methodological differences were identified in patient selection, specimen type, immunohistochemical protocols and positivity thresholds, reference molecular methods, mutation spectrum, and management of equivocal or failed results. Some cohorts were enriched for specific molecular profiles or used different units of analysis (patients, tumors, or tissue samples), introducing additional selection and verification bias. Consequently, pooling these non-equivalent comparisons could have produced clinically misleading summary estimates. Moreover, the number of studies within methodologically comparable subgroups was insufficient to support reliable stratified bivariate or HSROC analyses. Therefore, study-level sensitivity, specificity, concordance, and causes of discordance were synthesized descriptively.

We additionally considered whether a restricted bivariate or HSROC analysis could be performed using only unselected or consecutive cohorts evaluated on standardized automated staining platforms. However, this restriction did not yield a sufficiently homogeneous set of independent studies. The remaining reports continued to differ in specimen format, VE1 positivity thresholds, molecular reference methods and their analytical sensitivity, handling of equivocal results, molecular-verification strategy, and units of analysis. Moreover, the number of methodologically comparable studies providing complete paired diagnostic data was insufficient to support a stable hierarchical model. A restricted pooled analysis was therefore considered potentially misleading and was not performed.

ChatGPT (GPT-6) and Codex version 6 Astra (OpenAI, San Francisco, CA, USA; accessed July–August 2026) were used as assistive tools for calculation checking and preliminary preparation of tables and figures. They did not autonomously determine study eligibility, extract or adjudicate source data, perform risk-of-bias assessments, or determine the final interpretation of the evidence; all outputs were independently verified by the authors against the primary reports.

### 2.7. Risk of Bias Assessment

Methodological quality was assessed at the diagnostic-estimate level using QUADAS-3 [25]. The synthesis question was defined as follows: among patients with colorectal carcinoma, how accurately does VE1 immunohistochemistry identify molecularly confirmed BRAF p.V600E using paired testing of the same tumor specimen or a clearly defined overlapping cohort? The ideal diagnostic-accuracy study was defined a priori as an unselected consecutive or randomly sampled colorectal carcinoma cohort undergoing both VE1 immunohistochemistry and an appropriate molecular reference test, with prespecified interpretation criteria, blinded assessment, complete verification, transparent handling of equivocal results, and inclusion of all evaluable cases in the analysis.

When a report provided multiple estimates, one estimate corresponding most closely to the predefined synthesis question and intended clinical use was selected. No additional fixed hierarchy for selecting among multiple eligible estimates was prespecified. O.I.Ș. completed the assessment, and G.S. independently verified the signaling-question responses, domain-level judgments, applicability judgments, supporting evidence, and overall assessment. Disagreements were resolved by consensus. Risk of bias was evaluated in the Participants, Index test, Target condition, and Analysis domains, while applicability was evaluated for the first three domains. Overall risk-of-bias and applicability judgments were assigned in accordance with the QUADAS-3 guidance. Domain-level risk-of-bias and applicability judgments, together with their overall distributions, are provided in Supplementary Table S5A–C.

## 3. Results

### 3.1. Study Selection and Characteristics

**Figure 1:**
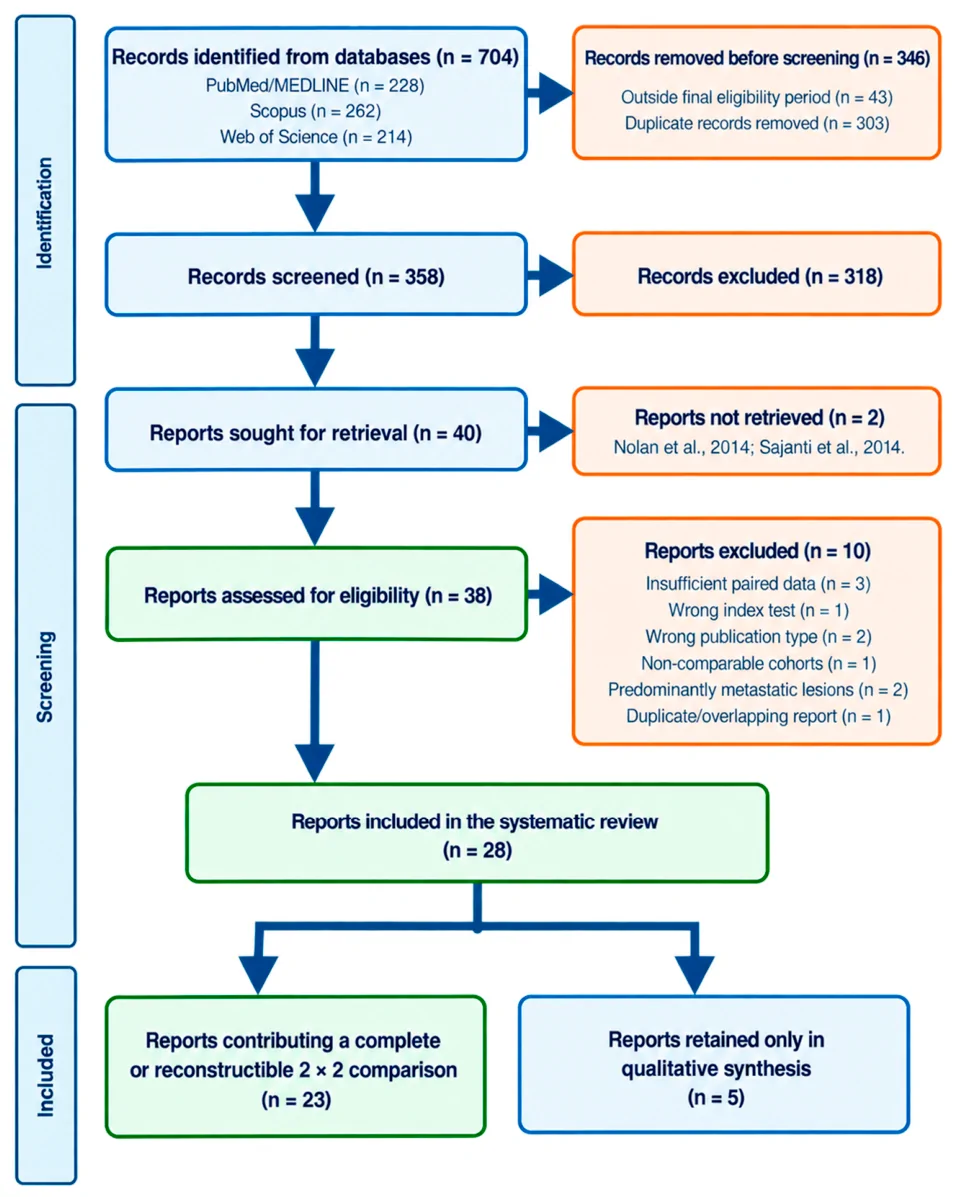
PRISMA 2020 flow diagram of study identification, screening, eligibility assessment, and inclusion [29,30].

The combined August 2025 searches and July 2026 update yielded 704 database records: 228 from PubMed/MEDLINE, 262 from Scopus, and 214 from Web of Science. Forty-three records outside the final eligibility period and 303 duplicate copies were removed, leaving 358 unique records for title/abstract screening. Of these, 318 were excluded and 40 database reports were sought for retrieval. Two database reports, Nolan et al. [29] and Sajanti et al. [30] passed title/abstract screening but could not be retrieved in full text. They were therefore recorded as not retrieved and were not subjected to full-text eligibility assessment, data extraction, or risk-of-bias assessment. Their non-inclusion represents a potential source of missing evidence, although its direction and magnitude cannot be determined. Thirty-eight full-text reports were assessed; ten were excluded for insufficient paired data (*n* = 3), wrong index test (*n* = 1), wrong publication type (*n* = 2), non-comparable cohorts (*n* = 1), predominantly metastatic lesions without an adequate primary-CRC analysis (*n* = 2), or duplicate/overlapping publication (*n* = 1). Named exclusions are reported in Supplementary Table S2 [3,8,19,21,31,32]. Twenty-eight reports were included [9,10,11,13,15,18,20,23,24,28,33,34,35,36,37,38,39,40,41,42,43,44,45,46,47,48,49,50]. The selection process is shown in Figure 1.

The included reports varied substantially in design and scale. Total CRC cohorts ranged from 19 cases in the colorectal subgroup of Loo et al. [35] to 990 unselected resections in Grillo et al. [49]. Several reports used mutation-enriched or genotype-selected samples [13,28,37,38,39,42,45], whereas others evaluated consecutive or routine cohorts [24,33,40,44,46,49]. Molecular comparators included Sanger sequencing, allele-specific or multiplex PCR, pyrosequencing, MassARRAY, cobas testing, quantitative PCR, and next-generation sequencing. In several reports, molecular verification was available only in an overlapping subset, which was used as the denominator for diagnostic comparisons [40,44,46,49].

Study-level technical characteristics varied considerably. The reports included whole-tissue sections, tissue microarrays, biopsies, and resection specimens; staining was performed on different automated or manual platforms using heterogeneous antigen-retrieval, detection, and interpretation protocols. Positivity criteria ranged from any cytoplasmic staining to prespecified combinations of staining intensity, distribution, and proportion of positive tumor cells. Equivocal staining was excluded, classified as positive or negative, or resolved by additional molecular testing, depending on the study. Molecular reference methods included Sanger sequencing, allele-specific PCR, pyrosequencing, SNaPshot, MassARRAY, cobas testing, quantitative PCR, and next-generation sequencing. Reporting of technical variables was frequently incomplete. Antigen-retrieval conditions, detailed interpretation criteria, reader number and experience, blinding status, and other processing parameters were not consistently reported across the included studies. This reporting deficiency limits direct protocol comparison and reduces the reproducibility and transferability of published VE1 performance estimates to routine laboratory practice.

### 3.2. Concordance Between VE1 Immunohistochemistry and Molecular Testing

Concordance was high in many optimized studies but was not uniform across protocols. Complete agreement was reported in small, selected cohorts such as Holck, Loo, Piton, and Dvorak (2019) [13,35,42,45], as well as in the 118 evaluable cases analyzed by Yambert et al. [48]. Larger routine-series evidence also supported high performance: Grillo et al. reported 99.1% sensitivity and 99.5% specificity after excluding equivocal score-1 cases from the binary calculation [49]. Conversely, Estrella et al. reported 74.6% sensitivity and 92.9% specificity with its Bond protocol [23], and Lasota et al. reported 85% sensitivity and 68% specificity [38].

Some reports could not be represented by a single unambiguous 2 × 2 matrix. Bledsoe et al. reported observer-averaged performance in a 165-case validation cohort [37]; Schafroth et al. reported observer-dependent sensitivity in a 34-case pilot cohort [41]; and Zhou et al. emphasized concordance and receiver-operating-characteristic performance after deriving new quantitative thresholds rather than reporting a conventional diagnostic matrix [50]. These studies were retained in qualitative synthesis, while their published metrics were not converted into invented cell counts. These characteristics and the principal concordance of the findings of the 28 eligible reports are summarized in Table 1. Reports retained in the qualitative synthesis without reconstructed 2 × 2 cell counts are listed in Supplementary Table S6.

### 3.3. Diagnostic Performance

**Figure 2:**
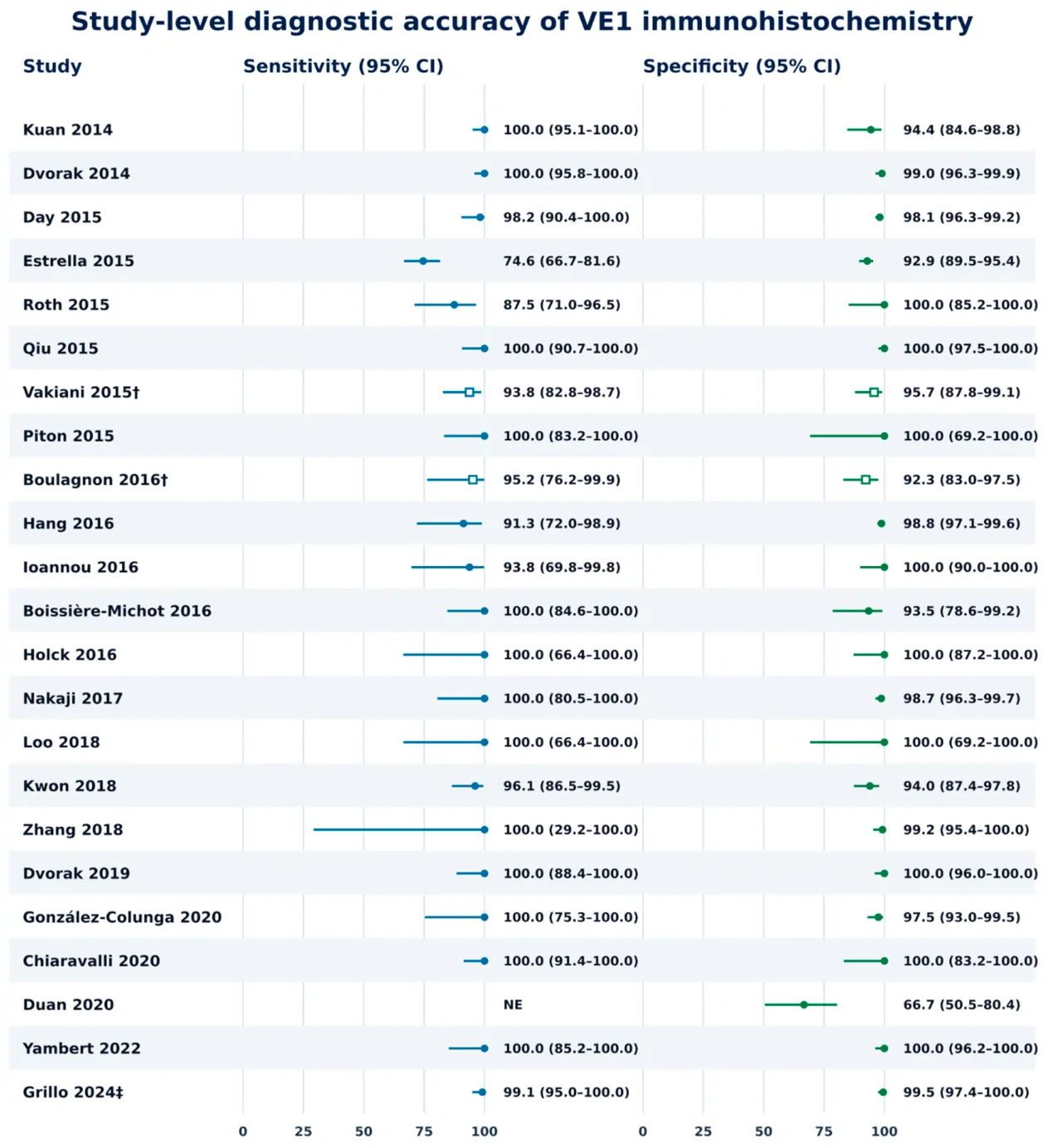
Study-level sensitivity and specificity of VE1 immunohistochemistry versus molecular testing, with exact 95% confidence intervals. Open squares (†) indicate equivocal results counted as discordant; ‡ indicates equivocal results excluded. NE, not estimable. No pooled estimate was calculated [9,10,11,13,15,18,20,23,24,28,33,34,35,37,38,39,40,41,42,43,44,45,46,47,48,49,50].

Among reports with published or derivable diagnostic estimates, sensitivity ranged from 74.6% to 100% and specificity from 66.7% to 100%. The lowest sensitivity was reported by Estrella et al. [23]. The lowest specificity occurred in the molecular-wild-type-only subset of Duan et al. (66.7%) [47], followed by the author-reported 68% specificity in Lasota et al. [38]. These results demonstrate that the VE1 clone alone does not guarantee reliable performance without complete protocol validation and explicit interpretation criteria. Study-level estimates and confidence intervals are displayed without pooling in Figure 2.

Study composition materially affected interpretation. Kuan, Kwon, Piton, Holck, Bledsoe, and both Dvorak studies used genotype-enriched or otherwise selected material [10,13,18,37,39,42,45]. Qiu et al. stained 611 CRCs but molecularly verified a 181-case subset [40]; Nakaji et al. stained 472 consecutive tumors but reported a 254-case paired sequencing subset [44]; and Grillo et al. performed IHC in 990 tumors with molecular results in 333 [49]. Predictive values and crude positive rates from these differently selected cohorts are therefore not directly comparable. Table 2 presents the complete/reconstructible matrices separately from author-reported metrics; exact 95% intervals are shown when a complete or clearly reconstructible 2 × 2 matrix was available. For Boulagnon et al. [9] and Vakiani et al. [28], equivocal results were conservatively classified as discordant; this approach can yield lower sensitivity and specificity estimates than analyses that exclude equivocal cases or resolve them through additional adjudication.

### 3.4. Findings in MLH1-Deficient Cohorts

Five reports provided particularly relevant evidence for Lynch syndrome screening or MLH1-deficient pathways (Table 3). Roth, Boissière-Michot, and Chiaravalli analyzed selected MLH1/PMS2-loss, MLH1-deficient MSI, or dMMR cohorts [20,34,36]. Yambert evaluated a combined MMR/BRAF IHC panel against NGS and obtained complete BRAF agreement in 118 evaluable cases [48]. Grillo evaluated an unselected 990-case series and reported 99.1% sensitivity and 99.5% specificity in the binary molecularly tested subset after excluding equivocal IHC results [49].

These studies support the operational use of VE1 as part of an MLH1-deficiency triage pathway, but they do not justify interpreting a negative VE1 result as evidence of Lynch syndrome. BRAF V600E positivity favors a sporadic MLH1-hypermethylated pathway; negative or equivocal results must be integrated with MLH1 promoter methylation, clinical/family history, and germline testing when indicated [20,34,36,48,49].

The available evidence derives predominantly from observational and routine-practice cohorts, with relatively limited prospective validation specifically designed to assess complete Lynch-syndrome screening pathways.

### 3.5. Technical and Interpretative Sources of Discordance

The most reproducible positive pattern was unequivocal cytoplasmic staining in tumor cells. Weak, focal, heterogeneous, nuclear, mucin-associated, or normal-crypt staining accounted for many discrepant or equivocal results. Bledsoe et al. showed that weak but diffuse and near-uniform staining may represent true positivity [37], whereas Vakiani et al. and Grillo et al. treated equivocal staining separately and required molecular clarification [28,49]. González-Colunga, Hang, and Kwon documented weak/focal false-positive or unstained mutant cases [10,24,33]. The practical implication is that laboratories must validate both intensity and distribution criteria rather than applying an intensity-only rule.

Specimen type and staining protocol also affected performance. Boulagnon et al. reported improved results when equivocal tissue-microarray cases were reviewed on whole sections [9]. Estrella et al. demonstrated substantial protocol-dependent discordance [23], whereas optimized automated protocols performed well in several later studies [40,45,48,49]. Zhou et al. further showed that previously published criteria produced only 80.4–84.8% initial concordance in its 250-case paired cohort; quantitatively optimized thresholds improved internal and external ROC performance, but atypical patterns still required molecular confirmation [50]. VE1 does not provide comprehensive BRAF genotyping and should not be expected to detect all non-V600E variants [35,46]. These results are shown in Table 4.

### 3.6. Risk of Bias and Applicability

**Figure 3:**
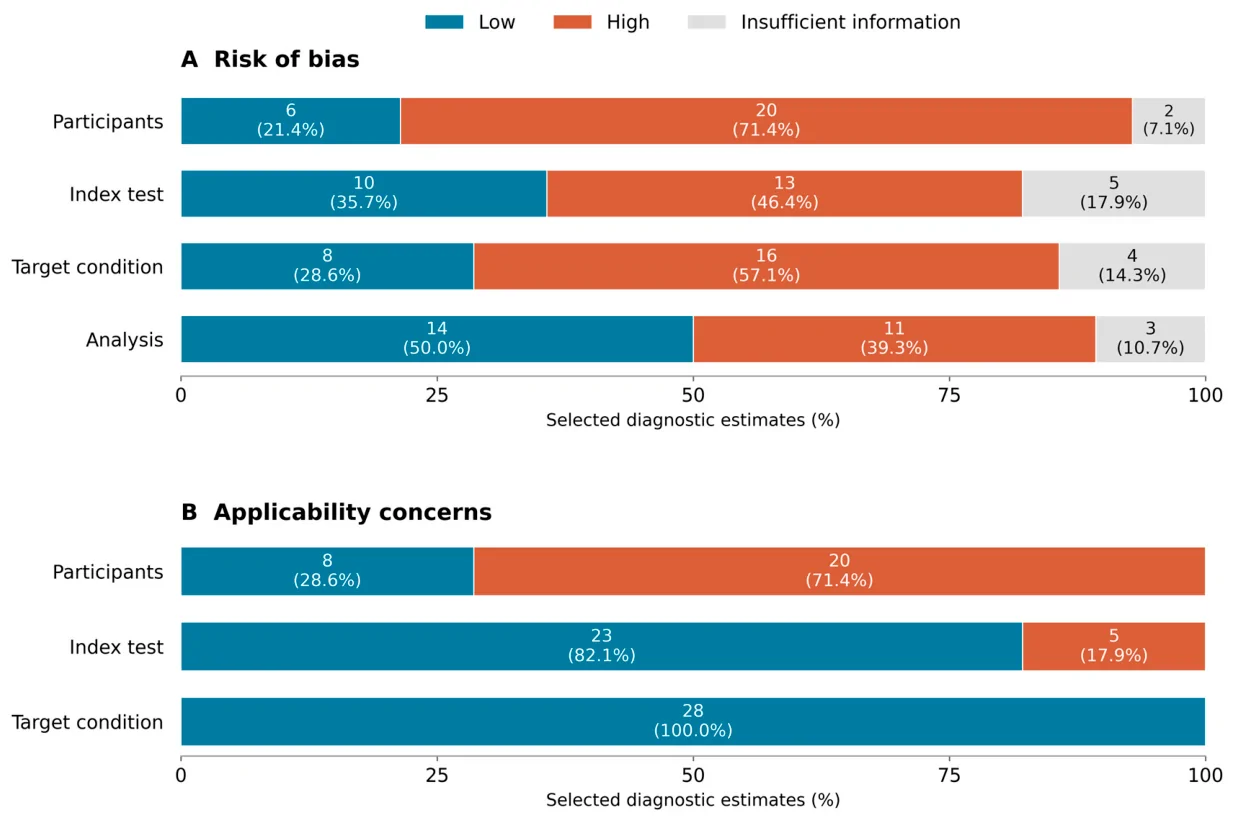
QUADAS-3 risk-of-bias (**A**) and applicability (**B**) judgments for 28 diagnostic estimates. Labels show counts and percentages; categories are low, high, and insufficient information.

The consensus QUADAS-3 assessment identified recurrent concerns rather than a uniformly low-risk evidence base. For Participants, 20 of 28 estimates were judged at high risk, 6 at low risk, and 2 as having insufficient information. For the Index test, 13 were high risk, 10 low risk, and 5 had insufficient information; for the Target condition, 16 were high risk, 8 low risk, and 4 had insufficient information; and for Analysis, 11 were high risk, 14 low risk, and 3 had insufficient information. High applicability concerns were concentrated in Participants (20 of 28 estimates); 5 estimates had high Index-test applicability concerns, and none had high Target-condition applicability concerns. The QUADAS-3 assessment demonstrated substantial methodological limitations across the evidence base. The principal concerns were genotype-enriched or otherwise selected cohorts, non-consecutive sampling, post hoc or incompletely defined VE1 thresholds, selective molecular verification, exclusion of equivocal results, and incomplete reporting of participant flow. Table 5 reports the domain-level and overall risk-of-bias judgments for each selected estimate, whereas Figure 3 presents the domain-level distribution of risk-of-bias and applicability judgments. Domain-level and overall distributions are provided in Supplementary Table S5A–C.

### 3.7. Suitability for Quantitative Synthesis

Twenty-three reports or defined subcohorts provided complete or clearly reconstructible 2 × 2 comparisons. Five reports contributed observer-specific, author-reported, or ROC-based evidence without a unique reconstructible matrix [37,38,41,46,50]. Among the reconstructible comparisons, substantial differences remained in cohort selection, specimen format, staining protocols, positivity thresholds, molecular reference methods, mutation spectrum, molecular-verification strategy, units of analysis, and handling of equivocal or failed results. The comparisons therefore did not provide a sufficiently comparable basis for pooling.

Restricting the evidence to unselected or consecutive cohorts evaluated on standardized automated platforms did not yield a sufficiently homogeneous set of independent studies with complete paired diagnostic data. Differences in specimen format, thresholds, reference-assay sensitivity, equivocal-result handling, verification strategy, and units of analysis persisted, and too few methodologically comparable studies remained to support a stable bivariate or HSROC model. We did not calculate a pooled estimate; we synthesized diagnostic performance and concordance descriptively.

## 4. Discussion

The final review includes 28 eligible reports identified after combining the August 2025 searches with the July 2026 update. Two additional reports could not be retrieved in full text and were not included in the evidence synthesis or QUADAS-3 assessment [29,30]. The amended 2014–2026 publication window captured both early VE1 validation studies and newly available evidence; the clinical and diagnostic eligibility criteria were otherwise unchanged. Across the included reports, VE1 positivity generally showed high agreement with molecular BRAF V600E testing when the staining protocol was optimized, and unequivocal cytoplasmic staining was required. However, the evidence also contains low-performing protocols, selectively verified cohorts, and non-binary equivocal categories [9,10,11,13,15,18,20,23,24,28,33,34,35,36,37,38,39,40,41,42,43,44,45,46,47,48,49,50].

The findings extend and qualify those of the 2016 meta-analysis by Pyo et al. [16]. That analysis summarized eight studies and reported high pooled diagnostic performance, whereas the present review includes 28 reports, including 12 published after 2016 and several substantially larger routine-practice cohorts. The expanded evidence base confirms that VE1 can perform well under optimized conditions but demonstrates that this performance is not uniform across clinical settings. Study-level sensitivity ranged from 74.6% to 100%, and specificity from 66.7% to 100%, with important variation related to cohort enrichment, tissue format, staining platform, positivity threshold, selective molecular verification, and handling of equivocal staining. Unlike the previous meta-analysis, the present review distinguishes complete paired comparisons from observer-dependent, selectively verified, or non-reconstructible estimates and evaluates risk of bias at the diagnostic-estimate level using QUADAS-3 (version 1.2). It also addresses the specific role of VE1 in MLH1-deficient colorectal carcinoma and Lynch syndrome triage. Thus, the principal added value of this review is the identification of the methodological and technical conditions under which previously reported high diagnostic accuracy may—or may not—be reproducible in clinical practice.

Several apparently perfect estimates arose from small or mutation-enriched cohorts [13,35,39,42,45], whereas larger routine cohorts generally produced narrow but non-zero discordance [33,40,44,49]. Such mutation-enriched or case-control designs may overestimate diagnostic performance relative to routine practice because they do not reproduce the prevalence and interpretative uncertainty encountered in consecutive clinical populations. Selective molecular verification in Qiu, Nakaji, Wedden, and Grillo means that their paired denominators are smaller than the IHC cohorts [40,44,46,49]. For this reason, cohort size, paired sample size, and handling of equivocal results are reported separately.

Selective molecular verification may also introduce partial-verification bias. In cohorts in which molecular confirmation was limited to selected cases or was preferentially performed in IHC-positive tumors, VE1-negative cases without molecular testing cannot be assumed to be true negatives. Consequently, false-negative VE1 results may remain undetected, potentially inflating apparent sensitivity and negative predictive value. Predictive values derived from selectively verified cohorts should therefore be interpreted cautiously and should not be considered equivalent to estimates obtained from complete paired molecular testing of both VE1-positive and VE1-negative tumors.

The most informative evidence came from reports that applied VE1 IHC staining and a molecular reference method to the same specimens or a clearly defined overlapping cohort. Twenty-three reports provided a complete or clearly reconstructible binary comparison; five others contributed observer-specific, author-reported, or ROC-based concordance evidence that could not be reduced safely to a unique 2 × 2 table [37,38,41,46,50]. Keeping those reports in the qualitative synthesis while withholding invented cell counts preserves relevant evidence without overstating numerical precision.

Most studies reported sensitivities and specificities above 90%, but clinically meaningful exceptions remained. Estrella et al. reported 74.6% sensitivity and 92.9% specificity [23]. Lasota et al. reported 85% sensitivity and 68% specificity [38], and Duan et al. obtained 66.7% specificity in a 42-case molecular-wild-type subset [47]. In contrast, recent large-scale evidence from Grillo et al. showed 99.1% sensitivity and 99.5% specificity after excluding equivocal score-1 results [49]. These contrasts indicate that use of VE1 must be validated as a complete laboratory protocol rather than accepted based on antibody clone alone.

Interpretation of weak, focal, or heterogeneous staining remains a central source of discordance. Weak diffuse staining may represent true positivity in some validated protocols [37], whereas weak focal, dot-like, nuclear, mucin-associated, or normal-crypt staining may be nonspecific [10,24,28,33,49]. This distinction is particularly important in small biopsies and tissue microarrays, in which limited sampling may not adequately represent heterogeneous tumor-cell expression. Weak, focal, heterogeneous, or otherwise equivocal staining in such limited specimens should therefore not be forced into a binary positive/negative classification. When additional tumor material is available, reassessment on a larger biopsy, whole-tissue section, or resection specimen should be considered, particularly when staining is heterogeneous or discordant with morphology or clinical findings. Persistently equivocal or clinically discordant results should undergo molecular confirmation using an appropriately sensitive assay. Accordingly, a stepwise approach is recommended: first reassess staining distribution, internal controls, tumor cellularity, and specimen adequacy; if the result remains equivocal, evaluate additional tumor tissue when available and proceed to molecular confirmation when uncertainty persists. An additional unresolved challenge is the absence of universally accepted criteria for defining equivocal VE1 staining, with substantial variation among laboratories in the intensity, distribution, and proportion thresholds used for classification.

Sampling and pre-analytical conditions also influence performance. Tissue microarrays can underrepresent heterogeneous staining, although small biopsies may perform well when tumor tissue is adequate and the protocol is validated [9,13,37]. Fixation, antigen retrieval, detection chemistry, platform, tumor cellularity, and interpretative training can each alter the result [18,23,37,39,45,49]. Cold ischemia time and detailed fixation conditions, including fixative type, buffering, and fixation duration, were inconsistently or incompletely reported across the source studies and therefore could not be compared quantitatively. This incomplete reporting limits the ability to distinguish discordance caused by the staining protocol itself from that related to pre-analytical tissue handling. The reproducibility achieved in some recent automated-platform studies should therefore be understood as protocol-specific rather than automatically transferable.

The molecular comparator and discordance pathway require equal attention. Included reports used Sanger sequencing, allele-specific and multiplex PCR, pyrosequencing, MassARRAY, cobas testing, qPCR, and NGS [9,10,11,13,15,18,20,23,24,28,33,34,35,36,37,38,39,40,41,42,43,44,45,46,47,48,49,50]. These assays are not analytically equivalent and differ in their ability to detect low mutant allele fractions. Consequently, particularly in specimens with low tumor cellularity, an apparently VE1-positive/molecular-negative result may reflect limited sensitivity of the initial molecular assay rather than a true immunohistochemical false positive. Accordingly, tumor cellularity and the analytical sensitivity of the reference assay should be considered before classifying a VE1-positive/molecular-negative case as an immunohistochemical false positive. Retesting discordant specimens with a more sensitive or orthogonal molecular method may therefore identify initially undetected BRAF p.V600E mutations. Such adjudicated results should nevertheless be reported separately from the initial comparison because they represent a different diagnostic assessment.

Evidence relevant to Lynch syndrome triage is supportive but conditional. In MLH1-deficient colorectal carcinoma, BRAF V600E positivity strongly favors a sporadic pathway and may reduce unnecessary germline referral [20,34,36,48,49]. A negative BRAF (VE1) result does not diagnose Lynch syndrome and must be integrated with MLH1 promoter methylation, clinical and family history, and germline assessment when indicated.

From an implementation perspective, VE1 offers rapid turnaround, direct visualization of the tumor-cell population, and accessibility in laboratories without routine molecular testing [10,15,20,49]. Roth et al. estimated a 10% institutional cost saving for an IHC-first strategy in its selected MLH1/PMS2-loss cohort [20]. This estimate is context-specific and should not be generalized across healthcare systems, but it illustrates the possible operational value of a validated triage pathway.

This review has several limitations. The protocol was not prospectively registered, and the publication window was expanded after the review began; the amendment therefore cannot be compared with a time-stamped protocol. Eligibility was restricted to English-language full-text articles, grey literature was not systematically searched, and two potentially relevant reports could not be retrieved. Study populations, positivity thresholds, tissue formats, molecular comparators, and handling of equivocal results were heterogeneous. Several reports verified only a subset molecularly, some used genotype-enriched samples, and five did not permit a unique 2 × 2 reconstruction. These limitations precluded an unqualified pooled estimate and require study-by-study interpretation. High-quality prospective multicentre studies evaluating VE1 within predefined real-world diagnostic pathways remain scarce, limiting assessment of reproducibility and clinical performance across institutions.

A further limitation of VE1 is biological: it targets the V600E mutant protein and does not provide comprehensive BRAF genotyping. Non-V600E alterations may remain immunohistochemically negative, as illustrated by reports containing alternative BRAF variants [35,46]. Molecular testing therefore remains necessary when comprehensive variant detection is clinically relevant or when the IHC result is equivocal, technically inadequate, or discordant with morphology and clinical context.

Taken together, the expanded evidence supports a conditional diagnostic role for BRAF (VE1 clone) immunohistochemistry in colorectal carcinoma. Reliable implementation requires validation of the complete staining protocol, explicit tumor-cell cytoplasmic criteria, appropriate controls, preservation of an equivocal category, and a defined pathway for molecular confirmation [9,10,15,18,23,24,28,33,37,45,49,50]. Under these conditions, VE1 clone can serve as an efficient first-line screen, including within selected Lynch syndrome screening algorithms, while molecular testing remains definitive for equivocal cases and comprehensive BRAF variant detection.

## 5. Conclusions

VE1 immunohistochemistry can serve as a rapid, locally validated triage assay for detecting BRAF p.V600E in colorectal carcinoma, but heterogeneity and recurrent risk-of-bias concerns do not support its universal substitution for molecular testing. Equivocal, technically inadequate, or clinically discordant results require molecular clarification, and molecular testing remains necessary when comprehensive BRAF genotyping or treatment-defining mutation assessment is required. Prospective multicentre validation using standardized interpretation criteria and complete paired molecular verification is needed to define the settings in which VE1 can reliably reduce molecular testing.

## Figures and Tables

**Table 1:** Characteristics and principal concordance findings of the 28 eligible reports.

Study	Study Population and Paired Cohort	Specimen/IHC Format	VE1 Interpretation or Protocol Feature	Molecular Reference	Verification and Equivocal-Result Handling
Bledsoe et al., 2014 [37]	204 CRC specimens; validation cohort *n* = 165; genotype-selected	CRC tissue; format NR	Observer-averaged interpretation	Clinical molecular testing	Validation metrics reported without a unique 2 × 2 matrix
Lasota et al., 2014 [38]	113 CRC specimens; 27 molecular V600E	NR	Study-specific interpretation criteria	Four molecular methods	Published totals did not permit unique reconstruction of the diagnostic matrix
Kuan et al., 2014 [39]	128 CRC specimens; retrospective and prospective selected cohorts	NR	Performance depended on rigorous protocol optimization	PCR-based testing	Three IHC-positive/molecular-wild-type cases
Dvorak et al., 2014 [18]	279-case CRC subset	NR	Binary VE1 interpretation	SNaPshot; NGS adjudication	Two unresolved IHC-positive/molecular-wild-type CRCs
Day et al., 2015 [15]	477 evaluable CRC specimens	Tissue microarray	Prespecified VE1 interpretation	Sanger sequencing; SNaPshot adjudication	Molecular adjudication was concentrated in discordant cases
Estrella et al., 2015 [23]	480 CRCs; paired binary cohort *n* = 465	Automated Leica Bond protocol; format NR	Protocol-dependent interpretation	Pyrosequencing, Sequenom and NGS	Fifteen non-V600E variants excluded from the V600E/wild-type matrix
Roth et al., 2015 [20]	55 MLH1/PMS2-deficient CRCs	NR	Binary positive/negative interpretation	Multiplex PCR	Selected Lynch-syndrome screening cohort
Qiu et al., 2015 [40]	611 IHC-tested CRCs; paired subset *n* = 181	NR	Binary VE1 interpretation	Sanger sequencing and real-time PCR	Only the 181-case subset underwent molecular verification
Schafroth et al., 2015 [41]	34-case diagnostic pilot cohort	NR	Interpretation varied between observers	Pyrosequencing	Observer-dependent evaluability prevented construction of a unique matrix
Vakiani et al., 2015 [28]	117 colorectal neoplasms	NR	Definite positive, negative and equivocal categories	MALDI-TOF; Sanger adjudication	Four equivocal cases retained separately; conservative reconstruction classified them as discordant
Piton et al., 2015 [42]	30 known-status CRC specimens	NR	Binary interpretation	SNaPshot	Mutation-enriched case-control cohort
Boulagnon et al., 2016 [9]	86 CRC specimens	Tissue microarrays and whole sections	Equivocal TMA staining reassessed on whole sections	TaqMan PCR; Sanger sequencing	Four equivocal TMA results classified as discordant in the conservative reconstruction
Hang et al., 2016 [33]	425 CRC specimens	Tissue microarray	Weak tumor-cell staining classified as positive	Sanger sequencing	Five weakly stained molecular-wild-type tumors
Ioannou et al., 2016 [43]	51 primary colon adenocarcinomas	NR	Binary interpretation; detailed threshold NR	DNA sequence analysis	Diagnostic matrix derived from the reported cohort data
Boissière-Michot et al., 2016 [34]	53 evaluable MLH1-deficient/MSI CRCs	NR	Binary VE1 interpretation	BRAF genotyping	Two IHC-positive/molecular-wild-type tumors
Holck et al., 2016 [13]	36 mutation-stratified CRC biopsies	Biopsy sections	Binary interpretation	cobas BRAF V600 test	Known-status, mutation-stratified cohort
Nakaji et al., 2017 [44]	472 IHC-tested CRCs; paired subset *n* = 254	NR	Binary interpretation	Direct sequencing	Selective molecular verification; three false-positive IHC results
Loo et al., 2018 [35]	Separately extractable 19-case CRC subgroup	NR	Binary interpretation	DNA sequencing	Complete agreement in the CRC subgroup
Kwon et al., 2018 [10]	151-case mutation-enriched cohort	NR	Nonspecific nuclear and weak staining considered during interpretation	Sequencing and allele-specific PCR	Mutation-enriched composition; six false-positive and two false-negative results
Zhang et al., 2018 [11]	123-case CRC subgroup	NR	Binary interpretation	ARMS-PCR; Sanger sequencing	One IHC-positive/molecular-wild-type tumor
Dvorak et al., 2019 [45]	120 CRC specimens	NR	Strict binary interpretation	Previous molecular testing; Sanger sequencing	Four equivocal KRAS-mutant/BRAF-wild-type cases were not classified as VE1-positive
Wedden et al., 2019 [46]	109 IHC-tested CRCs; molecularly verified *n* = 101	Routine clinical specimens	Routine-pathway interpretation	PCR and/or NGS	Eight IHC-tested tumors lacked molecular verification
González-Colunga et al., 2020 [24]	135 CRC specimens	NR	Weak or focal staining classified as positive	Pyrosequencing	Three weak/focal IHC-positive molecular-wild-type tumors
Chiaravalli et al., 2020 [36]	61 paired dMMR CRCs	NR	Binary interpretation	MassARRAY/Myriapod	Complete agreement in the paired cohort
Duan et al., 2020 [47]	42-case molecular-wild-type prediction subset	FFPE tissue sections	Weak and moderate cytoplasmic staining classified as positive	NGS	All 42 cases were molecularly wild type; only specificity was estimable
Yambert et al., 2022 [48]	126 sequenced CRCs; 118 evaluable	NR	Combined MMR/BRAF IHC interpretation	Clinical NGS	Eight cases were not included in the evaluable binary cohort
Grillo et al., 2024 [49]	990 IHC-tested CRCs; paired molecular subset *n* = 333	Unselected routine CRC series; format NR	Score-1 staining classified as equivocal	Molecular BRAF analysis	Twelve equivocal cases—3 mutant and 9 wild type—excluded from the reported binary estimate
Zhou et al., 2025 [50]	250 paired CRC specimens	NR	Quantitative thresholds developed using deep-learning-assisted analysis	qPCR and NGS	Post hoc optimized thresholds and ROC results; no single conventional diagnostic matrix

ARMS-PCR, amplification-refractory mutation system polymerase chain reaction; CRC, colorectal carcinoma; FFPE, formalin-fixed paraffin-embedded; IHC, immunohistochemistry; MSI, microsatellite instability; NGS, next-generation sequencing; NR, not reported; PCR, polymerase chain reaction; TMA, tissue microarray. Technical characteristics were recorded as NR when they were not explicitly reported and were not inferred.

**Table 2:** Diagnostic performance of VE1 immunohistochemistry against molecular testing.

Study	TP	FP	FN	TN	Sensitivity, % (95% CI)	Specificity, % (95% CI)	Qualification
Bledsoe et al., 2014 [37]	NR	NR	NR	NR	96.3 (reported)	98.5 (reported)	Observer-averaged validation cohort; no single matrix
Lasota et al., 2014 [38]	NR	NR	NR	NR	85.0 (reported)	68.0 (reported)	Published category totals do not yield a unique matrix
Kuan et al., 2014 [39]	74	3	0	51	100.0 (95.1–100.0)	94.4 (84.6–98.8)	Optimized protocol; selected cohort
Dvorak et al., 2014 [18]	87	2	0	190	100.0 (95.8–100.0)	99.0 (96.3–99.9)	CRC subgroup; two unresolved IHC false positives
Day et al., 2015 [15]	55	8	1	413	98.2 (90.4–100.0)	98.1 (96.3–99.2)	Initial sequencing comparison
Estrella et al., 2015 [23]	106	23	36	300	74.6 (66.7–81.6)	92.9 (89.5–95.4)	465 V600E/wild-type cases; non-V600E variants excluded
Roth et al., 2015 [20]	28	0	4	23	87.5 (71.0–96.5)	100.0 (85.2–100.0)	MLH1/PMS2-loss cohort
Qiu et al., 2015 [40]	38	0	0	143	100.0 (90.7–100.0)	100.0 (97.5–100.0)	Molecularly verified 181-case CRC subset
Schafroth et al., 2015 [41]	NR	NR	NR	NR	92.9–93.3 (reported)	100.0 (reported)	Observer-dependent evaluability; matrix not reconstructed
Vakiani et al., 2015 [28]	45	3	3	66	93.8 (82.8–98.7)	95.7 (87.8–99.1)	Conservative sensitivity analysis: four equivocal results counted as discordant
Piton et al., 2015 [42]	20	0	0	10	100.0 (83.2–100.0)	100.0 (69.2–100.0)	Known-status case-control cohort
Boulagnon et al., 2016 [9]	20	5	1	60	95.2 (76.2–99.9)	92.3 (83.0–97.5)	Conservative sensitivity analysis: four equivocal TMA results counted as discordant
Hang et al., 2016 [33]	21	5	2	397	91.3 (72.0–98.9)	98.8 (97.1–99.6)	Weak staining counted positive
Ioannou et al., 2016 [43]	15	0	1	35	93.8 (69.8–99.8)	100.0 (90.0–100.0)	Derived from published cohort and performance
Boissière-Michot et al., 2016 [34]	22	2	0	29	100.0 (84.6–100.0)	93.5 (78.6–99.2)	53 evaluable cases
Holck et al., 2016 [13]	9	0	0	27	100.0 (66.4–100.0)	100.0 (87.2–100.0)	Selected mutation-stratified biopsy cohort
Nakaji et al., 2017 [44]	17	3	0	234	100.0 (80.5–100.0)	98.7 (96.3–99.7)	Paired sequencing subset
Loo et al., 2018 [35]	9	0	0	10	100.0 (66.4–100.0)	100.0 (69.2–100.0)	CRC subgroup
Kwon et al., 2018 [10]	49	6	2	94	96.1 (86.5–99.5)	94.0 (87.4–97.8)	Enriched cohort
Zhang et al., 2018 [11]	3	1	0	119	100.0 (29.2–100.0)	99.2 (95.4–100.0)	CRC subgroup
Dvorak et al., 2019 [45]	30	0	0	90	100.0 (88.4–100.0)	100.0 (96.0–100.0)	Strict binary interpretation; four BRAF-WT equivocal cases not counted positive
Wedden et al., 2019 [46]	NR	NR	NR	NR	100.0 (reported)	100.0 (reported)	Complete V600E concordance in 101 verified cases; counts not separately tabulated
González-Colunga et al., 2020 [24]	13	3	0	119	100.0 (75.3–100.0)	97.5 (93.0–99.5)	Weak/focal staining counted positive
Chiaravalli et al., 2020 [36]	41	0	0	20	100.0 (91.4–100.0)	100.0 (83.2–100.0)	Paired dMMR subset
Duan et al., 2020 [47]	0	14	0	28	NE	66.7 (50.5–80.4)	Molecular-wild-type-only IHC subset; sensitivity not estimable
Yambert et al., 2022 [48]	23	0	0	95	100.0 (85.2–100.0)	100.0 (96.2–100.0)	118 evaluable cases; 100% overall agreement
Grillo et al., 2024 [49]	109	1	1	210	99.1 (95.0–100.0)	99.5 (97.4–100.0)	Published binary estimate excludes 12 equivocal cases (3 mutant; 9 wild type)
Zhou et al., 2025 [50]	NR	NR	NR	NR	NR	NR	Optimized criteria reported by ROC/AUC rather than a single matrix

CI, confidence interval; NE, not estimable; NR, not reported. For Boulagnon [9] and Vakiani [28], equivocal results were conservatively counted as discordant in the reconstructed matrix; Grillo [49] excluded 12 equivocal cases from its published binary estimate.

**Table 3:** VE1–molecular evidence relevant to Lynch syndrome screening and MLH1-deficient colorectal carcinoma.

Study	Paired Cohort	VE1-Positive	Molecular V600E	TP/FP/FN/TN	Sensitivity/Specificity (%)
Roth et al., 2015 [20]	55 MLH1/PMS2-loss	28	32	28/0/4/23	87.5/100
Boissière-Michot et al., 2016 [34]	53 MLH1-deficient MSI	24	22	22/2/0/29	100/93.5
Chiaravalli et al., 2020 [36]	61 paired dMMR	41	41	41/0/0/20	100/100
Yambert et al., 2022 [48]	118 evaluable	23	23	23/0/0/95	100/100
Grillo et al., 2024 [49]	333 molecularly tested	110 definite; 12 equivocal	113	109/1/1/210	99.1/99.5; equivocal excluded

**Table 4:** Technical and interpretative factors affecting VE1–molecular concordance [9,10,11,13,15,18,20,23,24,28,33,34,35,36,37,38,39,40,41,42,43,44,45,46,47,48,49,50].

Factor	Verified Finding	Implication
Antibody specificity	VE1 targets the BRAF V600E mutant protein and does not provide comprehensive BRAF genotyping.	A negative VE1 result cannot exclude non-V600E BRAF variants.
Positive pattern	Unequivocal tumor-cell cytoplasmic staining is the most reproducible positive pattern.	Validate intensity and distribution criteria locally.
Equivocal patterns	Weak focal, heterogeneous, nuclear, dot-like, mucin-associated, or normal-crypt staining may be nonspecific.	Retain an equivocal category and confirm molecularly.
Weak diffuse staining	Some optimized protocols identify weak but diffuse/near-uniform staining as true positive.	Do not use intensity alone; consider distribution and controls.
Tissue sampling	TMA, biopsy, and whole-section sampling may yield different results.	Reassess weak, focal, heterogeneous, or equivocal staining on a larger biopsy or whole section when additional tissue is available
Tumor cellularity/specimen adequacy	Limited viable tumor content may compromise reliable interpretation and contribute to IHC–molecular discordance.	Confirm adequate tumor representation before interpreting equivocal or discordant results and assess additional tissue when available.
Platform and retrieval	Performance varied across Leica Bond and Ventana protocols and with retrieval/detection chemistry.	Validate the complete protocol, not the clone alone.
Reference method	Sanger, PCR-based assays, pyrosequencing, MassARRAY, cobas, qPCR, and NGS have different analytical sensitivities.	Consider tumor cellularity and assay sensitivity when interpreting discordant results; use an appropriately sensitive orthogonal molecular method for clinically important discordance.
Verification strategy	Several studies molecularly tested only a subset of IHC-stained tumors.	Report paired denominators, avoid treating unverified cases as true negatives, and interpret sensitivity and NPV cautiously when VE1-negative cases were not systematically verified.
Equivocal exclusion	Excluding equivocal cases may improve binary sensitivity/specificity.	Report equivocal counts separately and define their clinical pathway.
Study design	Mutation-enriched, MLH1-deficient, or genotype-selected cohorts alter prevalence and spectrum.	Do not compare predictive values directly across differently selected cohorts.

**Table 5:** Consensus QUADAS-3 risk-of-bias judgments for the 28 selected diagnostic estimates.

Study	Participants	Index Test	Target Condition	Analysis	Overall Risk of Bias
Bledsoe et al., 2015 [37]	High	High	High	High	High
Lasota et al., 2014 [38]	High	High	Low	Insufficient information	High
Kuan et al., 2014 [39]	High	High	Insufficient information	Low	High
Dvorak et al., 2014 [18]	Insufficient information	High	High	Low	High
Day et al., 2015 [15]	Low	Low	High	High	High
Estrella et al.1, 2015 [23]	High	High	High	High	High
Roth et al., 2015 [20]	High	Low	Low	Low	High
Qiu et al., 2015 [40]	High	Insufficient information	Insufficient information	Low	High
Schafroth et al., 2015 [41]	High	High	High	Insufficient information	High
Vakiani et al., 2015 [28]	High	Low	High	High	High
Piton et al., 2015 [42]	High	Low	Low	Low	High
Boulagnon et al., 2016 [9]	High	Low	Low	Low	High
Hang et al., 2016 [33]	Low	High	Insufficient information	Low	High
Ioannou et al., 2016 [43]	High	Low	Low	Low	High
Boissière-Michot et al., 2016 [34]	High	Low	High	High	High
Holck et al., 2016 [13]	High	High	Low	Low	High
Nakaji et al., 2017 [44]	Low	Low	High	High	High
Loo et al., 2018 [35]	High	High	High	Low	High
Kwon et al., 2018 [10]	High	High	High	Low	High
Zhang et al., 2018 [11]	Low	Low	Low	Low	Low
Dvorak et al., 2019 [45]	High	Insufficient information	High	Low	High
Wedden et al., 2019 [46]	Low	Insufficient information	High	High	High
González-Colunga et al., 2020 [24]	Low	High	Low	Low	High
Chiaravalli et al., 2020 [36]	High	Insufficient information	High	High	High
Duan et al., 2020 [47]	High	Insufficient information	High	High	High
Yambert et al., 2022 [48]	High	Low	High	High	High
Grillo et al., 2024 [49]	High	High	High	High	High
Zhou et al., 2025 [50]	Insufficient information	High	Insufficient information	Insufficient information	High

Low = low risk of bias; High = high risk of bias; Insufficient = insufficient information.

## Data Availability

The study-level evidence used in this review was extracted from published reports. Additional review materials, including the screening audit and detailed QUADAS-3 assessment records, are available from the corresponding author on reasonable request.

## Supplementary Materials

The following supporting information can be downloaded at: https://www.mdpi.com/article/10.3390/medicina62101974/s1, Table S1, Search, screening, retrieval, and inclusion counts; Table S2, Reports excluded after full-text assessment; Table S3, Reports sought but not retrieved; Table S4, Prespecified handling and reporting of equivocal VE1 results; Table S5A–C, QUADAS-3 risk-of-bias and applicability assessments; Table S6, Reports retained in the qualitative synthesis without reconstructed 2 × 2 cell counts. PRISMA 2020 Checklist.

## Abbreviations

The following abbreviations are used in this manuscript:

ARMS-PCR	Amplification-refractory mutation system polymerase chain reaction
AUC	Area under the receiver-operating-characteristic curve
BRAF	B-Raf proto-oncogene, serine/threonine kinase
CI	Confidence interval
CRC	Colorectal cancer
dMMR	Deficient mismatch repair
DNA	Deoxyribonucleic acid
EGFR	Epidermal growth factor receptor
ERK	Extracellular signal-regulated kinase
FFPE	Formalin-fixed, paraffin-embedded
FN	False negative
FP	False positive
IHC	Immunohistochemistry
MAPK	Mitogen-activated protein kinase
MEK	Mitogen-activated protein kinase kinase
MLH1	MutL homolog 1
MMR	Mismatch repair
MSI	Microsatellite instability
MSI-H	Microsatellite instability-high
NGS	Next-generation sequencing
NPV	Negative predictive value
OPA	Overall percent agreement
PCR	Polymerase chain reaction
PMS2	PMS1 homolog 2, mismatch repair system component
PPV	Positive predictive value
PRISMA	Preferred Reporting Items for Systematic Reviews and Meta-Analyses
qPCR	Quantitative polymerase chain reaction
QUADAS-3	Quality Assessment of Diagnostic Accuracy Studies 3
RAF	Rapidly accelerated fibrosarcoma kinase
RAS	Rat sarcoma virus proto-oncogene family
ROC	Receiver-operating-characteristic
TMA	Tissue microarray
TN	True negative
TP	True positive
V600E	Substitution of valine by glutamic acid at codon 600
VE1	BRAF V600E mutation-specific monoclonal antibody clone

## References

[1] Tudorache M., Treteanu A.-R., Gradisteanu Pircalabioru G., Lixandru-Petre I.-O., Bolocan A., Andronic O. (2025). Gut Microbiome Alterations in Colorectal Cancer: Mechanisms, Therapeutic Strategies, and Precision Oncology Perspectives. Cancers.

[2] Morgan E., Arnold M., Gini A., Lorenzoni V., Cabasag C.J., Laversanne M., Vignat J., Ferlay J., Murphy N., Bray F. (2023). Global Burden of Colorectal Cancer in 2020 and 2040: Incidence and Mortality Estimates from GLOBOCAN. Gut.

[3] Wu J.-B., Li X.-J., Liu H., Liu Y.-J., Liu X.-P. (2023). Association of KRAS, NRAS, BRAF and PIK3CA Gene Mutations with Clinicopathological Features, Prognosis and Ring Finger Protein 215 Expression in Patients with Colorectal Cancer. Biomed. Rep..

[4] Awad H., Elshebli S., Hasan K., Eid Y., Obeidat F., Alzyoud M., Alakhras B., AlShammas F. (2024). Comparing Clinicopathological and Immunohistochemical Features of Colorectal Carcinoma between Young and Old Age Groups. Diagnostics.

[5] Sung H., Filho A.M., Laversanne M., Ferlay J., Siegel R.L., Soerjomataram I., Jemal A., Bray F. (2026). Global Cancer Statistics 2024: GLOBOCAN Estimates of Incidence and Mortality Worldwide for 34 Cancers in 186 Countries. CA Cancer J. Clin..

[6] Patel M., McSorley S.T., Park J.H., Roxburgh C.S.D., Edwards J., Horgan P.G., McMillan D.C. (2018). The Relationship between Right-Sided Tumour Location, Tumour Microenvironment, Systemic Inflammation, Adjuvant Therapy and Survival in Patients Undergoing Surgery for Colon and Rectal Cancer. Br. J. Cancer.

[7] Kasprzak A. (2023). Prognostic Biomarkers of Cell Proliferation in Colorectal Cancer (CRC): From Immunohistochemistry to Molecular Biology Techniques. Cancers.

[8] Hassan S., Mirza T., Khatoon A., Bukhari U., Shaikh F., Karim A. (2024). BRAF Mutations and the Association of V600E with CD133 and CDX2 Expression in a Pakistani Colorectal Carcinoma Cohort. BMC Cancer.

[9] Boulagnon C., Dudez O., Beaudoux O., Dalstein V., Kianmanesh R., Bouché O., Diebold M.-D. (2016). BRAF V600E Gene Mutation in Colonic Adenocarcinomas. Immunohistochemical Detection Using Tissue Microarray and Clinicopathologic Characteristics: An 86 Case Series. Appl. Immunohistochem. Mol. Morphol..

[10] Kwon J.-H., Jeong B.-K., Yoon Y.S., Yu C.S., Kim J. (2018). Utility of BRAF VE1 Immunohistochemistry as a Screening Tool for Colorectal Cancer Harboring BRAF V600E Mutation. J. Pathol. Transl. Med..

[11] Zhang X., Wang L., Wang J., Zhao H., Wu J., Liu S., Zhang L., Li Y., Xing X. (2018). Immunohistochemistry Is a Feasible Method to Screen BRAF V600E Mutation in Colorectal and Papillary Thyroid Carcinoma. Exp. Mol. Pathol..

[12] Bläker H., Alwers E., Arnold A., Herpel E., Tagscherer K.E., Roth W., Jansen L., Walter V., Kloor M., Chang-Claude J. (2019). The Association Between Mutations in BRAF and Colorectal Cancer–Specific Survival Depends on Microsatellite Status and Tumor Stage. Clin. Gastroenterol. Hepatol..

[13] Holck S., Bonde J., Pedersen H., Petersen A.A., Chaube A., Nielsen H.J., Larsson L.-I. (2016). Localization of Active, Dually Phosphorylated Extracellular Signal-Regulated Kinase 1 and 2 in Colorectal Cancer with or without Activating BRAF and KRAS Mutations. Hum. Pathol..

[14] Lee M.K.C., Loree J.M. (2019). Current and Emerging Biomarkers in Metastatic Colorectal Cancer. Curr. Oncol..

[15] Day F., Muranyi A., Singh S., Shanmugam K., Williams D., Byrne D., Pham K., Palmieri M., Tie J., Grogan T. (2015). A Mutant BRAF V600E-Specific Immunohistochemical Assay: Correlation with Molecular Mutation Status and Clinical Outcome in Colorectal Cancer. Targ. Oncol..

[16] Pyo J.-S., Sohn J.H., Kang G. (2016). Diagnostic Accuracy of BRAF Immunohistochemistry in Colorectal Cancer: A Meta-Analysis and Diagnostic Test Accuracy Review. Pathol. Oncol. Res..

[17] Cremolini C., Chalabi M., Elez E., Fassan M., Gelli M., Goéré D., Modest D.P., Montagut C., Osterlund P., Ricke J. (2026). Metastatic Colorectal Cancer: ESMO Clinical Practice Guideline for Diagnosis, Treatment and Follow-Up. Ann. Oncol..

[18] Dvorak K., Aggeler B., Palting J., McKelvie P., Ruszkiewicz A., Waring P. (2014). Immunohistochemistry with the Anti-BRAF V600E (VE1) Antibody: Impact of Pre-Analytical Conditions and Concordance with DNA Sequencing in Colorectal and Papillary Thyroid Carcinoma. Pathology.

[19] Galuppini F., Pennelli G., Loupakis F., Lanza C., Vianello L., Sacchi D., Mescoli C., Salmaso R., Agostini M., Lonardi S. (2017). *BRAF* p.V600E-specific Immunohistochemical Assessment in Colorectal Cancer Endoscopy Biopsies Is Consistent with the Mutational Profiling. Histopathology.

[20] Roth R.M., Hampel H., Arnold C.A., Yearsley M.M., Marsh W.L., Frankel W.L. (2015). A Modified Lynch Syndrome Screening Algorithm in Colon Cancer. Am. J. Clin. Pathol..

[21] Zhang X., Wu J., Wang L., Zhao H., Li H., Duan Y., Li Y., Xu P., Ran W., Xing X. (2020). *HER2* and *BRAF* Mutation in Colorectal Cancer Patients: A Retrospective Study in Eastern China. PeerJ.

[22] Saizul Z., Siti-Azrin A.H., Zakaria A.D., Hassan A., Abdul Rahman W.F.W., Jalil N.A.C. (2021). BRAF V600E and Mismatch Repair Proteins Expression in Sporadic Young-Onset Colorectal Cancer in Kelantan, Malaysia. Oman Med. J..

[23] Estrella J.S., Tetzlaff M.T., Bassett R.L., Patel K.P., Williams M.D., Curry J.L., Rashid A., Hamilton S.R., Broaddus R.R. (2015). Assessment of *BRAF* V600E Status in Colorectal Carcinoma: Tissue-Specific Discordances between Immunohistochemistry and Sequencing. Mol. Cancer Ther..

[24] González-Colunga K.J., Lino-Silva L.S., Salcedo-Hernández R.A., Ruiz-García E.B., Zepeda-Najar C. (2020). BRAF V600E Expression by Immunohistochemistry in Colon Cancer and Clinico-Pathologic Features Associated with BRAF-Mutated Colonic Cancers in Mexican Patients. J. Gastrointest. Cancer.

[25] Whiting P.F., Tomlinson E., Rutjes A.W.S., Davenport C.F., Yang B., Westwood M.E., Takwoingi Y., Reitsma J.B., Hyde C., Bossuyt P.M.M. (2026). QUADAS-3: A Revised Tool for the Quality Assessment of Diagnostic Test Accuracy Studies. Ann. Intern. Med..

[26] Page M.J., McKenzie J.E., Bossuyt P.M., Boutron I., Hoffmann T.C., Mulrow C.D., Shamseer L., Tetzlaff J.M., Akl E.A., Brennan S.E. (2021). The PRISMA 2020 Statement: An Updated Guideline for Reporting Systematic Reviews. BMJ.

[27] McInnes M.D.F., Moher D., Thombs B.D., McGrath T.A., Bossuyt P.M., Clifford T., Cohen J.F., Deeks J.J., Gatsonis C., the PRISMA-DTA Group (2018). Preferred Reporting Items for a Systematic Review and Meta-Analysis of Diagnostic Test Accuracy Studies: The PRISMA-DTA Statement. JAMA.

[28] Vakiani E., Yaeger R., Brooke S., Zhou Y., Klimstra D.S., Shia J. (2015). Immunohistochemical Detection of the BRAF V600E Mutant Protein in Colorectal Neoplasms. Appl. Immunohistochem. Mol. Morphol..

[29] Nolan S., Arnason T., Drucker A., Huang W.-Y. (2014). The Utility of BRAFV600E Mutation-Specific Antibody for Colon Cancers With Microsatellite Instability. Appl. Immunohistochem. Mol. Morphol..

[30] Sajanti S., Sirniö P., Väyrynen J.P., Tuomisto A., Klintrup K., Mäkelä J., Ristimäki A., Mäkinen M.J. (2014). VE1 Immunohistochemistry Accurately Detects BRAF V600E Mutations in Colorectal Carcinoma and Can Be Utilized in the Detection of Poorly Differentiated Colorectal Serrated Adenocarcinoma. Virchows Arch..

[31] Deng W., Liu X., Huang S., Wu Z., Alessandro F., Lin Q., Cai Z., Zhang Z., Huang Y., Wang H. (2024). CXCL16 Promotes Tumor Metastasis by Regulating Angiogenesis in the Tumor Micro-Environment of BRAF V600E Mutant Colorectal Cancer. Transl. Oncol..

[32] Lee K.S., Kwak Y., Ahn S., Shin E., Oh H.-K., Kim D.-W., Kang S.-B., Choe G., Kim W.H., Lee H.S. (2017). Prognostic Implication of CD274 (PD-L1) Protein Expression in Tumor-Infiltrating Immune Cells for Microsatellite Unstable and Stable Colorectal Cancer. Cancer Immunol. Immunother..

[33] Hang J., Li A.F., Chang S., Liang W. (2016). Immunohistochemical Detection of the *BRAF* V600E Mutant Protein in Colorectal Cancers in Taiwan Is Highly Concordant with the Molecular Test. Histopathology.

[34] Boissière-Michot F., Frugier H., Ho-Pun-Cheung A., Lopez-Crapez E., Duffour J., Bibeau F. (2016). Immunohistochemical Staining for P16 and BRAFV600E Is Useful to Distinguish between Sporadic and Hereditary (Lynch Syndrome-Related) Microsatellite Instable Colorectal Carcinomas. Virchows Arch..

[35] Loo E., Khalili P., Beuhler K., Siddiqi I., Vasef M.A. (2018). BRAF V600E Mutation Across Multiple Tumor Types: Correlation Between DNA-Based Sequencing and Mutation-Specific Immunohistochemistry. Appl. Immunohistochem. Mol. Morphol..

[36] Chiaravalli A.M., Carnevali I., Sahnane N., Leoni E., Furlan D., Berselli M., Sessa F., Tibiletti M.G. (2020). Universal Screening to Identify Lynch Syndrome: Two Years of Experience in a Northern Italian Center. Eur. J. Cancer Prev..

[37] Bledsoe J.R., Kamionek M., Mino-Kenudson M. (2014). BRAF V600E Immunohistochemistry Is Reliable in Primary and Metastatic Colorectal Carcinoma Regardless of Treatment Status and Shows High Intratumoral Homogeneity. Am. J. Surg. Pathol..

[38] Lasota J., Kowalik A., Wasag B., Wang Z.-F., Felisiak-Golabek A., Coates T., Kopczynski J., Gozdz S., Miettinen M. (2014). Detection of the BRAF V600E Mutation in Colon Carcinoma: Critical Evaluation of the Imunohistochemical Approach. Am. J. Surg. Pathol..

[39] Kuan S.-F., Navina S., Cressman K.L., Pai R.K. (2014). Immunohistochemical Detection of BRAF V600E Mutant Protein Using the VE1 Antibody in Colorectal Carcinoma Is Highly Concordant with Molecular Testing but Requires Rigorous Antibody Optimization. Hum. Pathol..

[40] Qiu T., Lu H., Guo L., Huang W., Ling Y., Shan L., Li W., Ying J., Lv N. (2015). Detection of BRAF Mutation in Chinese Tumor Patients Using a Highly Sensitive Antibody Immunohistochemistry Assay. Sci. Rep..

[41] Schafroth C., Galván J.A., Centeno I., Koelzer V.H., Dawson H.E., Sokol L., Rieger G., Berger M.D., Hädrich M., Rosenberg R. (2015). VE1 Immunohistochemistry Predicts *BRAF* V600E Mutation Status and Clinical Outcome in Colorectal Cancer. Oncotarget.

[42] Piton N., Borrini F., Bolognese A., Lamy A., Sabourin J.-C. (2015). *KRAS* and *BRAF* Mutation Detection: Is Immunohistochemistry a Possible Alternative to Molecular Biology in Colorectal Cancer?. Gastroenterol. Res. Pract..

[43] Ioannou M., Papamichali R., Samara M., Paraskeva E., Papacharalambous C., Baxevanidou K., Koukoulis G. (2016). Diagnostic Value of Immunohistochemistry for the Detection of the BRAF V600E Mutation in Colorectal Carcinoma. J. BUON.

[44] Nakaji Y., Oki E., Nakanishi R., Ando K., Sugiyama M., Nakashima Y., Yamashita N., Saeki H., Oda Y., Maehara Y. (2017). Prognostic Value of BRAF V600E Mutation and Microsatellite Instability in Japanese Patients with Sporadic Colorectal Cancer. J. Cancer Res. Clin. Oncol..

[45] Dvorak K., Higgins A., Palting J., Cohen M., Brunhoeber P. (2019). Immunohistochemistry with Anti-BRAF V600E (VE1) Mouse Monoclonal Antibody Is a Sensitive Method for Detection of the BRAF V600E Mutation in Colon Cancer: Evaluation of 120 Cases with and without KRAS Mutation and Literature Review. Pathol. Oncol. Res..

[46] Wedden S., Miller K., Frayling I.M., Thomas T., Chefani A., Miller K., Hamblin A., Taylor J.C., D’Arrigo C. (2019). Colorectal Cancer Stratification in the Routine Clinical Pathway: A District General Hospital Experience. Appl. Immunohistochem. Mol. Morphol..

[47] Duan J., Yang Y., Yin L., Zhang X., Tang Y., Zhang S., Gong H., Xiao M., Li M., Li Q. (2020). Preliminary Study on the Identification of BRAFV600E Mutation in Colorectal Cancer by Near-Infrared Spectroscopy. OncoTargets Ther..

[48] Yambert J., Henricksen L.A., Clements J., Hannon A., Jordan A., Singh S., Dvorak K., Pritchard C.C., Konnick E.Q. (2022). DNA Mismatch Repair Proteins and BRAF V600E Detection by Immunohistochemistry in Colorectal Cancer Demonstrates Concordance with Next Generation Sequencing. J. Mol. Pathol..

[49] Grillo F., Paudice M., Pigozzi S., Dono M., Lastraioli S., Lugaresi M., Bozzano S., Tognoni C., Ali M., Sciallero S. (2024). BRAF^V600E^ Immunohistochemistry Can Reliably Substitute *BRAF* Molecular Testing in the Lynch Syndrome Screening Algorithm in Colorectal Cancer. Histopathology.

[50] Zhou Y., Li J., Zhou C., Hou J., Wang J., Lan T., Wan D., Tu Y., Chen Y., Yang Q. (2025). Deep Learning–Guided Quantitative Analysis Establishes Optimized BRAF V600E Immunohistochemical Criteria for Colorectal Cancer: A Multiplatform Validation Study. Lab. Investig..

