# Supplementary material for: Diagnostic Accuracy of VE1 Immunohistochemistry for BRAF p.V600E in Colorectal Carcinoma: A Systematic Review
Source: Medicina (Kaunas). 2026 Oct 12;62(10):1974. doi: 10.3390/medicina62101974 (PMC13706911; doi:10.3390/medicina62101974)
Supplement: Supplementary file 1 [file medicina-62-01974-s001.zip › medicina-4527648-supplementary.pdf]

medicina

MDPI

Supplementary Material

Diagnostic Accuracy of VE1 Immunohistochemistry for BRAF p.V600E in Colorectal Carcinoma: A Systematic Review

Ovidiu-Ioan Șușu [1,2,3], Marian Botoncea [1,2,*], Gabriel Serac [2], Marius-Alexandru Beleaua [1,4], Tamás-Csaba Sipos [1,5], Oana Alexandra Tăurean [1], Dragoș Călin Molnar [2], Remus-Sebastian Șipoș [1] and Zsuzsánna Pap [1]

1 Faculty of Medicine, George Emil Palade University of Medicine, Pharmacy, Sciences and Technology of Târgu Mureș, 38 Gheorghe Marinescu Street, 540139 Târgu Mureș, Romania; ovidiu.susu@umfst.ro (O.-I.Ș.); marius.beleaua@umfst.ro (M.-A.B.); tamas.sipos@umfst.ro (T.-C.S.); oanataurean@yahoo.com (O.A.T.); remus.sipos@umfst.ro (R.-S.Ș.); zsuzsanna.pap@umfst.ro (Z.P.)
2 General Surgery Clinic No. 1, County Emergency Clinical Hospital of Târgu Mureș, 50 Gheorghe Marinescu Street, 540136 Târgu Mureș, Romania; gabiserac@gmail.com (G.S.); dragosmolnar2000@yahoo.com (D.C.M.)
3 Doctoral School of Medicine and Pharmacy, George Emil Palade University of Medicine, Pharmacy, Sciences and Technology of Târgu Mureș, 540142 Târgu Mures, Romania
4 Pathology Department, County Emergency Clinical Hospital of Bistrița, 43 General Grigore Bălan Boulevard, 420094 Bistrița-Năsăud, Romania
5 Pathology Department, County Emergency Clinical Hospital of Târgu Mureș, 50 Gheorghe Marinescu Street, 540136 Târgu Mureș, Romania
* Correspondence: marian.botoncea@umfst.ro; Tel.: +40-758-468-909

Purpose. This file provides the report-level audit details supporting the PRISMA flow diagram, the descriptive diagnostic synthesis, and the estimate-level QUADAS-3 assessment. Search counts correspond to the combined August 2025 searches and the 27 July 2026 update.

Table S1. Search, screening, retrieval, and inclusion counts.

Stage	n	Clarification
Database records identified	704	PubMed/MEDLINE 228; Scopus 262; Web of Science 214
Records outside final publication window	43	Removed before screening
Duplicate copies removed	303	DOI, PMID, exact-title, and normalized-title matching with manual review
Unique records screened	358	Title/abstract screening
Records excluded	318	Title/abstract stage
Database reports sought	40	Full-text retrieval stage
Reports not retrieved	2	Nolan; Sajanti
Full-text reports assessed	38	Retrieved database reports assessed for eligibility
Full-text reports excluded	10	One primary exclusion reason per report
Reports/studies included	28	One included report per study
Complete or clearly reconstructible 2 × 2 comparisons	23	Included in the unpooled forest plot when sensitivity or specificity was estimable
Qualitative/author-reported diagnostic evidence	5	No unique 2 × 2 matrix

Table S2. Reports excluded after full-text assessment (n = 10).

Report	Primary reason	Report-level justification	Identifier
Wu et al., 2023	Insufficient paired data	Molecular BRAF results combined V600E with other V600 variants; a V600E-specific paired matrix could not be reconstructed.	10.3892/br.2023.1686
Hassan et al., 2024	Wrong index test	The mutation-specific antibody was clone RM8 rather than VE1.	10.1186/s12885-024-12925-z
Galuppini et al., 2017	Wrong publication type	The report was explicitly published as correspondence.	10.1111/his.13315
Zhang et al., 2020	Insufficient paired data	Molecular testing was limited to IHC-positive cases; IHC-negative cases were not molecularly verified.	10.7717/peerj.8602
Deng et al., 2024	Insufficient paired data	No extractable paired diagnostic counts; the antibody clone was not specified.	10.1016/j.tranon.2023.101854
Lee et al., 2017	Non-comparable cohorts	Molecular testing was limited to IHC-positive/equivocal MSI-H tumors.	10.1007/s00262-017-1999-6
Løes et al., 2015	Predominantly metastatic lesions	The CRC material comprised 383 liver metastases and only 6 primary tumors; the IHC comparison subset was overwhelmingly metastatic.	10.1007/s13277-014-2711-5
Ilie et al., 2014	Predominantly metastatic lesions	The study evaluated thoracic metastases from 310 patients with metastatic CRC and did not provide an adequate primary-CRC cohort.	10.1097/PAT.0000000000000113
Zhang et al., meeting abstract	Wrong publication type	Only abstract 864 in a Modern Pathology annual-meeting supplement was located; no eligible full article was identified.	Annual-meeting abstract
Hang et al., meeting abstract	Duplicate/overlapping report	Preliminary report of the full Hang et al. article retained in the review.	Annual-meeting abstract

Table S3. Reports sought but not retrieved (n = 2).

Report	Title	DOI	Review handling
Nolan et al., 2014	The Utility of BRAFV600E Mutation-Specific Antibody for Colon Cancers with Microsatellite Instability	10.1097/PAI.0000000000000026	Not assessed, extracted, or included in QUADAS-3
Sajanti et al., 2014	VE1 Immunohistochemistry Accurately Detects BRAF V600E Mutations in Colorectal Carcinoma and Can Be Utilized in the	10.1007/s00428-014-1555-0	Not assessed, extracted, or

Report	Title	DOI	Review handling
	Detection of Poorly Differentiated Colorectal Serrated Adenocarcinoma		included in QUADAS-3

Table S4. Prespecified handling and reporting of equivocal VE1 results.

Report	Published equivocal information	Matrix used in Table 2	Review handling
Boulagnon et al., 2016	22 definite IHC-positive; 4 equivocal TMA results	TP 20; FP 5; FN 1; TN 60	Conservative sensitivity analysis counted molecular-positive equivocal as FN and molecular-negative equivocal as FP; whole-section review was described separately.
Vakiani et al., 2015	46 definite IHC-positive; 4 equivocal results	TP 45; FP 3; FN 3; TN 66	Conservative sensitivity analysis counted equivocal results as discordant; the row is flagged and is not treated as a standard published binary estimate.
Dvorak et al., 2019	4 equivocal KRAS-mutant/BRAF-wild-type cases	TP 30; FP 0; FN 0; TN 90	Equivocal cases were not counted positive in the strict binary comparison.
Grillo et al., 2024	12 score-1 equivocal cases: 3 mutant and 9 wild type	TP 109; FP 1; FN 1; TN 210	The published binary estimate excluded the 12 equivocal cases; this exclusion is stated in the main table and forest-plot note.

QUADAS-3 assessment

One primary diagnostic estimate was assessed per included report. Reports without a uniquely reconstructible 2 × 2 table were retained as author-reported/qualitative estimates. Ovidiu-Ioan Șușu completed the source-linked assessment; Marian Botoncea independently verified the signaling responses, risk-of-bias judgments, applicability judgments, and supporting evidence. Differences were resolved by discussion and consensus on 4 August 2026. Overall risk of bias was rated high when at least one domain was high, low when all four domains were low, and insufficient when no domain was high and at least one domain had insufficient information. Overall applicability was derived analogously across the three applicability domains. No numerical quality score was used.

Table S5A. Distribution of QUADAS-3 domain-level judgments across 28 included reports.

Assessment	Domain	Low, n (%)	High, n (%)	Insufficient, n (%)
Risk of bias	Participants	6 (21.4)	20 (71.4)	2 (7.1)
Risk of bias	Index test	10 (35.7)	13 (46.4)	5 (17.9)
Risk of bias	Target condition	8 (28.6)	16 (57.1)	4 (14.3)
Risk of bias	Analysis	14 (50.0)	11 (39.3)	3 (10.7)
Applicability	Participants	8 (28.6)	20 (71.4)	0 (0.0)
Applicability	Index test	23 (82.1)	5 (17.9)	0 (0.0)
Applicability	Target condition	28 (100.0)	0 (0.0)	0 (0.0)

Table S5B. Estimate-level QUADAS-3 risk-of-bias judgments.

Study	Ref.	Estimate	Participants	Index test	Target condition	Analysis	Overall
Boulagnon et al., 2016	9	Paired binary	High	Low	Low	Low	High
Kwon et al., 2018	10	Paired binary	High	High	High	Low	High
Zhang et al., 2018	11	Paired binary	Low	Low	Low	Low	Low
Holck et al., 2016	13	Paired binary	High	High	Low	Low	High
Day et al., 2015	15	Paired binary	Low	Low	High	High	High
Roth et al., 2015	20	Paired binary	High	Low	Low	Low	High
Estrella et al., 2015	23	Paired binary	High	High	High	High	High
González-Colunga et al., 2020	24	Paired binary	Low	High	Low	Low	High
Hang et al., 2016	33	Paired binary	Low	High	Insufficient information	Low	High
Boissière-Michot et al., 2016	34	Paired binary	High	Low	High	High	High
Loo et al., 2018	35	Paired binary	High	High	High	Low	High
Chiaravalli et al., 2020	36	Paired binary	High	Insufficient information	High	High	High
Bledsoe et al., 2014	37	Author-reported/qualitative	High	High	High	High	High
Lasota et al., 2014	38	Author-reported/qualitative	High	High	Low	Insufficient information	High
Kuan et al., 2014	39	Paired binary	High	High	Insufficient information	Low	High
Dvorak et al., 2014	18	Paired binary	Insufficient information	High	High	Low	High
Qiu et al., 2015	40	Paired binary	High	Insufficient information	Insufficient information	Low	High
Schafroth et al., 2015	41	Author-reported/qualitative	High	High	High	Insufficient information	High
Vakiani et al., 2015	28	Paired binary	High	Low	High	High	High
Piton et al., 2015	42	Paired binary	High	Low	Low	Low	High
Ioannou et al., 2016	43	Paired binary	High	Low	Low	Low	High
Nakaji et al., 2017	44	Paired binary	Low	Low	High	High	High

https://doi.org/10.3390/medicina62101974

Study	Ref.	Estimate	Participants	Index test	Target condition	Analysis	Overall
Dvorak et al., 2019	45	Paired binary	High	Insufficient information	High	Low	High
Wedden et al., 2019	46	Author-reported/qualitative	Low	Insufficient information	High	High	High
Duan et al., 2020	47	Paired binary	High	Insufficient information	High	High	High
Yambert et al., 2022	48	Paired binary	High	Low	High	High	High
Grillo et al., 2024	49	Paired binary	High	High	High	High	High
Zhou et al., 2025	50	Author-reported/qualitative	Insufficient information	High	Insufficient information	Insufficient information	High

Table S5C. Estimate-level QUADAS-3 applicability judgments.

Study	Ref.	Participants	Index test	Target condition	Overall
Boulagnon et al., 2016	9	High	Low	Low	High
Kwon et al., 2018	10	High	Low	Low	High
Zhang et al., 2018	11	Low	Low	Low	Low
Holck et al., 2016	13	High	Low	Low	High
Day et al., 2015	15	Low	Low	Low	Low
Roth et al., 2015	20	High	Low	Low	High
Estrella et al., 2015	23	High	Low	Low	High
González-Colunga et al., 2020	24	Low	Low	Low	Low
Hang et al., 2016	33	Low	High	Low	High
Boissière-Michot et al., 2016	34	High	Low	Low	High
Loo et al., 2018	35	High	Low	Low	High
Chiaravalli et al., 2020	36	High	Low	Low	High
Bledsoe et al., 2014	37	High	High	Low	High
Lasota et al., 2014	38	High	High	Low	High
Kuan et al., 2014	39	High	Low	Low	High
Dvorak et al., 2014	18	Low	High	Low	High
Qiu et al., 2015	40	High	Low	Low	High
Schafroth et al., 2015	41	High	High	Low	High
Vakiani et al., 2015	28	High	Low	Low	High
Piton et al., 2015	42	High	Low	Low	High
Ioannou et al., 2016	43	High	Low	Low	High
Nakaji et al., 2017	44	Low	Low	Low	Low
Dvorak et al., 2019	45	High	Low	Low	High
Wedden et al., 2019	46	Low	Low	Low	Low
Duan et al., 2020	47	High	Low	Low	High
Yambert et al., 2022	48	High	Low	Low	High
Grillo et al., 2024	49	High	Low	Low	High
Zhou et al., 2025	50	Low	Low	Low	Low

Table S6. Reports retained in the qualitative synthesis without invented 2 × 2 cell counts.

Report	Reason a unique matrix was not reconstructed
Bledsoe et al., 2014	Observer-averaged validation metrics; no single report-level matrix
Lasota et al., 2014	Published category totals did not reconcile to one unique binary matrix
Schafroth et al., 2015	Observer-dependent sensitivity and evaluability
Wedden et al., 2019	Complete concordance reported for 101 verified cases without separately tabulated cells
Zhou et al., 2025	ROC/AUC-based threshold optimization rather than one conventional binary matrix

Abbreviations: AUC, area under the curve; CI, confidence interval; CRC, colorectal carcinoma; FN, false negative; FP, false positive; IHC, immunohistochemistry; MSI-H, microsatellite instability-high; NGS, next-generation sequencing; QUADAS-3, Quality Assessment of Diagnostic Accuracy Studies, version 3; ROC, receiver operating characteristic; ROB, risk of bias; TMA, tissue microarray; TN, true negative; TP, true positive.

https://doi.org/10.3390/medicina62101974

PRISMA 2020 Checklist

Diagnostic Accuracy of VE1 Immunohistochemistry for BRAF p.V600

E in Colorectal Carcinoma: A Systematic Review

Topic	Item	Checklist item	Location in manuscript
Title	**1**	Identify the report as a systematic review.	Title page
Abstract	**2**	See the PRISMA 2020 for Abstracts checklist.	Abstract
Rationale	**3**	Describe the rationale for the review in the context of existing knowledge.	Section 1
Objectives	**4**	Provide an explicit statement of the objective(s) or question(s) the review addresses.	Section 1, final paragraphs
Eligibility criteria	**5**	Specify the inclusion and exclusion criteria for the review and how studies were grouped for the syntheses.	Sections 2.3 and 2.6
Information sources	**6**	Specify all databases, registers, websites, organisations, reference lists and other sources searched or consulted to identify studies. Specify the date when each source was last searched or consulted.	Section 2.2
Search strategy	**7**	Present the full search strategies for all databases, registers and websites, including any filters and limits used.	Section 2.2
Selection process	**8**	Specify the methods used to decide whether a study met the inclusion criteria of the review, including how many reviewers screened each record and each report retrieved, whether they worked independently, and if applicable, details of automation tools used in the process.	Section 2.4
Data collection process	**9**	Specify the methods used to collect data from reports, including how many reviewers collected data from each report, whether they worked independently, any processes for obtaining or confirming data from study investigators, and if applicable, details of automation tools used in the process.	Section 2.5
Data items	**10a**	List and define all outcomes for which data were sought. Specify whether all results that were compatible with each outcome domain in each study were sought (e.g. for all measures, time points, analyses), and if not, the methods used to decide which results to collect.	Sections 2.5–2.6
Data items	**10b**	List and define all other variables for which data were sought (e.g. participant and intervention characteristics, funding sources). Describe any assumptions made about any missing or unclear information.	Section 2.5
Study risk of bias assessment	**11**	Specify the methods used to assess risk of bias in the included studies, including details of the tool(s) used, how many reviewers assessed each study and whether they worked independently, and if applicable, details of automation tools used in the process.	Section 2.7
Effect measures	**12**	Specify for each outcome the effect measure(s) (e.g. risk ratio, mean difference) used in the synthesis or presentation of results.	Section 2.6
Synthesis methods	**13a**	Describe the processes used to decide which studies were eligible for each synthesis (e.g. tabulating the study intervention characteristics and comparing against the planned groups for each synthesis (item #5)).	Sections 2.3, 2.6
Synthesis methods	**13b**	Describe any methods required to prepare the data for presentation or synthesis, such as handling of missing summary statistics, or data conversions.	Section 2.6
Synthesis methods	**13c**	Describe any methods used to tabulate or visually display results of individual studies and syntheses.	Sections 2.6 and 3.2–3.6; Tables 1–5; Figures 2–3
Synthesis methods	**13d**	Describe any methods used to synthesize results and provide a rationale for the choice(s). If meta-analysis was performed, describe the model(s), method(s) to identify the presence and extent of statistical heterogeneity, and software package(s) used.	Section 2.6 (descriptive synthesis; meta-analysis not performed)
Synthesis methods	**13e**	Describe any methods used to explore possible causes of heterogeneity among study results (e.g. subgroup analysis, meta-regression).	Section 2.6 (narrative examination); Sections 3.2–3.6
Synthesis methods	**13f**	Describe any sensitivity analyses conducted to assess robustness of the synthesized results.	Section 2.6 (conservative classification of equivocal results); Table 2
Reporting bias assessment	**14**	Describe any methods used to assess risk of bias due to missing results in a synthesis (arising from reporting biases).	No separate formal method reported; limitations discussed in Section 4
Certainty assessment	**15**	Describe any methods used to assess certainty (or confidence) in the body of evidence for an outcome.	No formal certainty-of-evidence assessment reported

Topic	Item	Checklist item	Location in manuscript
Study selection	**16a**	Describe the results of the search and selection process, from the number of records identified in the search to the number of studies included in the review, ideally using a flow diagram.	Section 3.1; Figure 1; Supplementary Table S1
Study selection	**16b**	Cite studies that might appear to meet the inclusion criteria, but which were excluded, and explain why they were excluded.	Section 3.1; Supplementary Tables S2–S3
Study characteristics	**17**	Cite each included study and present its characteristics.	Sections 3.1–3.2; Table 1
Risk of bias in studies	**18**	Present assessments of risk of bias for each included study.	Section 3.6; Table 5; Figure 3; Supplementary Table S5A–C
Results of individual studies	**19**	For all outcomes, present, for each study: (a) summary statistics for each group (where appropriate) and (b) an effect estimate and its precision (e.g. confidence/credible interval), ideally using structured tables or plots.	Section 3.3; Table 2; Figure 2
Results of syntheses	**20a**	For each synthesis, briefly summarise the characteristics and risk of bias among contributing studies.	Sections 3.2–3.6; Tables 1 and 5
Results of syntheses	**20b**	Present results of all statistical syntheses conducted. If meta-analysis was done, present for each the summary estimate and its precision (e.g. confidence/credible interval) and measures of statistical heterogeneity. If comparing groups, describe the direction of the effect.	Not applicable: no meta-analysis; Section 2.6
Results of syntheses	**20c**	Present results of all investigations of possible causes of heterogeneity among study results.	Sections 3.2, 3.3, 3.5; Section 4
Results of syntheses	**20d**	Present results of all sensitivity analyses conducted to assess the robustness of the synthesized results.	Section 3.3; Table 2 (conservative equivocal classification)
Reporting biases	**21**	Present assessments of risk of bias due to missing results (arising from reporting biases) for each synthesis assessed.	No formal assessment reported; Section 4 discusses missing evidence
Certainty of evidence	**22**	Present assessments of certainty (or confidence) in the body of evidence for each outcome assessed.	No formal certainty-of-evidence assessment reported
Discussion	**23a**	Provide a general interpretation of the results in the context of other evidence.	Section 4
Discussion	**23b**	Discuss any limitations of the evidence included in the review.	Section 4
Discussion	**23c**	Discuss any limitations of the review processes used.	Section 4
Discussion	**23d**	Discuss implications of the results for practice, policy, and future research.	Sections 4–5
Registration and protocol	**24a**	Provide registration information for the review, including register name and registration number, or state that the review was not registered.	Section 2.1 (not prospectively registered)
Registration and protocol	**24b**	Indicate where the review protocol can be accessed, or state that a protocol was not prepared.	*Protocol availability not explicitly stated in manuscript; Section 2.1*
Registration and protocol	**24c**	Describe and explain any amendments to information provided at registration or in the protocol.	Section 2.1 (revised publication window); Section 4
Support	**25**	Describe sources of financial or non-financial support for the review, and the role of the funders or sponsors in the review.	Funding statement; Acknowledgments
Competing interests	**26**	Declare any competing interests of review authors.	Conflicts of Interest statement
Availability of data and materials	**27**	Report which of the following are publicly available and where they can be found: template data collection forms; data extracted from included studies; data used for all analyses; analytic code; any other materials used in the review.	Data Availability Statement; Supplementary Tables S1–S6

From: Page MJ, McKenzie JE, Bossuyt PM, Boutron I, Hoffmann TC, Mulrow CD, et al. The PRISMA 2020 statement: an updated guideline for reporting systematic reviews. BMJ 2021;372:n71. doi: 10.1136/bmj.n71. This work is licensed under CC BY 4.0. To view a copy of this license, visit https://creativecommons.org/licenses/by/4.0/
